# Effectiveness of systemic family therapy versus treatment as usual for young people after self-harm: a pragmatic, phase 3, multicentre, randomised controlled trial

**DOI:** 10.1016/S2215-0366(18)30058-0

**Published:** 2018-03

**Authors:** David J Cottrell, Alexandra Wright-Hughes, Michelle Collinson, Paula Boston, Ivan Eisler, Sarah Fortune, Elizabeth H Graham, Jonathon Green, Allan O House, Michael Kerfoot, David W Owens, Eirini-Christina Saloniki, Mima Simic, Fiona Lambert, Justine Rothwell, Sandy Tubeuf, Amanda J Farrin

**Affiliations:** a Leeds Institute of Health Sciences, University of Leeds, Leeds, UK; b Clinical Trials Research Unit, University of Leeds, Leeds, UK; c Department of Child and Adolescent Psychiatry, Institute of Psychiatry, Psychology and Neurological Science, Kings College London, London, UK; d Division of Neuroscience and Experimental Psychology, School of Biological Sciences, University of Manchester, Manchester, UK; e South London and Maudsley NHS Foundation Trust, London, UK; f Royal Manchester Children's Hospital, Manchester, UK; g Centre for Health Services Studies and Personal Social Services Research Unit, University of Kent, Canterbury, UK

## Abstract

**Background:**

Self-harm in adolescents is common and repetition occurs in a high proportion of these cases. Scarce evidence exists for effectiveness of interventions to reduce self-harm.

**Methods:**

This pragmatic, multicentre, randomised, controlled trial of family therapy versus treatment as usual was done at 40 UK Child and Adolescent Mental Health Services (CAMHS) centres. We recruited young people aged 11–17 years who had self-harmed at least twice and presented to CAMHS after self-harm. Participants were randomly assigned (1:1) to receive manualised family therapy delivered by trained and supervised family therapists or treatment as usual by local CAMHS. Participants and therapists were aware of treatment allocation; researchers were masked. The primary outcome was hospital attendance for repetition of self-harm in the 18 months after group assignment. Primary and safety analyses were done in the intention-to-treat population. The trial is registered at the ISRCTN registry, number ISRCTN59793150.

**Findings:**

Between Nov 23, 2009, and Dec 31, 2013, 3554 young people were screened and 832 eligible young people consented to participation and were randomly assigned to receive family therapy (n=415) or treatment as usual (n=417). Primary outcome data were available for 795 (96%) participants. Numbers of hospital attendances for repeat self-harm events were not significantly different between the groups (118 [28%] in the family therapy group *vs* 103 [25%] in the treatment as usual group; hazard ratio 1·14 [95% CI 0·87–1·49] p=0·33). Similar numbers of adverse events occurred in both groups (787 in the family therapy group *vs* 847 in the treatment as usual group).

**Interpretation:**

For adolescents referred to CAMHS after self-harm, having self-harmed at least once before, our family therapy intervention conferred no benefits over treatment as usual in reducing subsequent hospital attendance for self-harm. Clinicians are therefore still unable to recommend a clear, evidence-based intervention to reduce repeated self-harm in adolescents.

**Funding:**

National Institute for Health Research Health Technology Assessment programme.

## Introduction

Self-harm in adolescents is a global public health problem, with 10% of adolescents self-reporting self-harm within the past year^1^ and suicide the second commonest cause of death in young people aged 10–24 years, after road traffic accidents.^2^ Self-harm in adolescents has serious consequences, and those who self-harm have a four times greater risk of death from any cause and a ten times greater risk of suicide than the general population,^2^, ^3^, ^4^ indicating potentially avoidably high burdens of life-years lost and family and peer distress. Non-fatal repetition occurs in 18% of people who self-harm, according to a recent large multicentre study in England.^5^

A single effective intervention has not been identified.^6^ A recent systematic review and meta-analysis of 19 randomised controlled trials with 2176 participants found a small overall effect of three specific interventions (dialectical behaviour therapy, mentalisation-based therapy, and cognitive behavioural therapy) on repetition of self-harm.^7^ Studies with strong family involvement and substantial treatment dose showed significant reductions in self-harm events.^7^, ^8^, ^9^ A recent large, retro-spective, registry-based matched cohort study (n=5678) showed lower long-term risk of self-harm in people receiving psychosocial treatments compared with those who did not, but numbers needed to treat were large.^10^

Family factors (parent–child interaction, perceived support, expressed emotion, experience of abuse, parental conflict, and parental mental health) are important risk factors associated with self-harm in children and adolescents.^11^ Family therapy aims to draw on and mobilise the existing strengths and resources of the child and family and is therefore a logical potential intervention after self-harm.^12^

**Research in context**

**Evidence before this study**

We searched electronic databases Embase, MEDLINE, PsycINFO, and the Cochrane Database of Systematic Reviews for randomised controlled trials of interventions to address self-harm in people younger than 18 years in which the primary outcome was reduction in self-harm. We included trials published up to March 31, 2007, in any language. Because of the varied nomenclature used in self-harm research, our search used several keywords for self-harm and associated behaviours as follows: “self-harm” OR “deliberate self-harm” OR “suicide” OR “attempted suicide” OR “overdose” OR “suicidal behaviour” OR “drug overdose” OR “self-poisoning” OR “self-injurious behaviour” OR “self-injury” OR, “non-suicidal self-injury” OR “self-destructive behaviour” OR “self-inflicted wounds” OR “self-mutilation” OR “suicidal ideation”. We screened abstracts to retrieve full-text articles for assessment of eligibility, and checked reference lists of relevant studies and reviews for additional references.

We identified one trial of a token allowing readmission to hospital, which found no effect, and one trial of group therapy for adolescents, but no other studies in young people (aged 18 years or younger) with a primary outcome of reduction in repetition of self-harm (subsequent replication of the group therapy study did not find a positive effect of group therapy). We identified two studies of family interventions related to self-harm, a study in people with depression that reported suicidal ideation as a secondary outcome, and a study of a home-based intervention designed to improve family communication, powered to detect between-group differences in suicidal ideation, not repeat self-harm.

**Added value of the study**

We found no evidence that, for adolescents referred to Child and Adolescent Mental Health Services (CAMHS) for self-harm, having self-harmed at least once before, the trial's manualised systemic family therapy conferred any benefits over treatment as usual in reducing subsequent hospital admission for self-harm. Interpretation of health economic and secondary outcomes was limited by significant loss to follow-up, but our data suggest possible significant improvements in secondary clinical outcomes, such as extent of emotional and behavioural problems, and the possibility of cost-effectiveness when considering combined benefits to the caregiver and young person together.

**Interpretation**

For adolescents referred to CAMHS after self-harm, having self-harmed at least once before, SHIFT family therapy conferred no benefits over treatment as usual in reducing subsequent hospital attendance for self-harm. Young people who self-harm form a varied and heterogeneous group, and self-harm is likely to be the final common pathway for a wide range of problems. Further research is needed to develop a more personalised approach and to identify which interventions are most helpful for which young people.

This trial, termed the Self-Harm Intervention: Family Therapy (SHIFT) trial, reports on a new form of family therapy intervention for self-harm. The trial was done in response to a call by the National Institute for Health Research Health Technology Assessment programme for a study investigating the clinical effectiveness and cost-effectiveness of family therapy for adolescents who self-harm (HTA 07/33). We aimed to assess the effectiveness of family therapy compared with treatment as usual in reducing self-harm repetition in young people.

## Methods

### Study design and participants

This study is a pragmatic, multicentre, individually randomised, controlled trial of family therapy versus treatment as usual, done at 40 UK National Health Service (NHS) Child and Adolescent Mental Health Services (CAMHS) in 15 NHS trusts in the UK across Greater Manchester, London, and Yorkshire. The study was approved by the UK NHS National Research Ethics Service in April, 2009 (09/H1307/20), and the protocol is published online.^13^

Eligible adolescents were aged 11–17 years, living with a primary caregiver (who was willing to take part), and had self-harmed at least twice before being referred to CAMHS for self-harm (index episode). If the self-harm event was caused by alcohol or recreational drugs, the young person had to have stated that they were intending self-harm by use of these substances. In common with UK, European, and Australian practice,^2^ we defined self-harm as any form of intentional non-fatal self-poisoning or self-injury (eg, cutting, taking excess medication, hanging, self-strangulation, jumping from height, and running into traffic) regardless of suicidal intent; this includes US definitions of non-suicidal self-injury and suicidal behaviour. Exclusion criteria were serious risk of suicide, an ongoing child protection investigation in the family, pregnancy at time of trial entry, usual treatment by a specific specialist service within CAMHS, residence in a short-term foster home, moderate to severe learning disabilities, involvement in another study within the 6 months before entry into this trial, sibling participation in the trial or treatment with family therapy within CAMHS, and insufficient proficiency in English language of either the young person or caregiver to complete study questionnaires (appendix). All patients and carers gave written informed consent to participate in the trial.

### Randomisation and masking

Participants were randomly assigned sequentially to receive family therapy or treatment as usual (1:1) via a computer-generated minimisation programme incorpor-ating a random element. Stratification factors were centre (CAMHS teams); sex; age (11–14 years or 15–17 years); living arrangements (with parents or guardians *vs* long-term foster care); previous self-harm episodes (two *vs* at least three); and index episode type (self-poisoning, self-injury, or combination). Family therapists working across multiple CAMHS (15 CAMHS in Manchester, two in London, and four in Yorkshire) were randomly allocated to family therapy participants. Participants and therapists were aware of treatment allocation, whereas researchers were masked to enable unbiased follow-up.

### Procedures

The research funder commissioned an analysis of a family therapy intervention for self-harm in people aged 11–17 years. Justifications for this are provided elsewhere^11^, ^12^, ^13^ and in the appendix. Young people were screened by a clinician at CAMHS after the index self-harm episode. Patients eligible and consenting to researcher contact were visited at home by a researcher to discuss the trial, obtain written consent for participation, and conduct baseline assessments.

The family therapy intervention^14^ was based on a modified version of an existing manual,^15^ allowing flexibility to deliver a complex intervention by experienced, qualified, family therapists able to make sophisticated clinical judgements.^16^ SHIFT family therapists received initial and ongoing training and monthly 2-h group supervision with a senior trial supervisor (PB, IE, or Charlotte Burke [Tavistock and Portman NHS Foundation Trust]).

Family therapy sessions lasted about 1·25 h and were delivered over 6 months at approximately monthly intervals, though more frequently initially. An intervention of six to eight sessions was selected after discussions with clinical services suggested that this would be the maximum number of sessions that could be delivered within usual resources. These discussions, alongside national audit data,^17^ indicated that typical intervention length in UK CAMH services was five to eight sessions. Therapist adherence to family therapy was ensured through training, use of the manual, and regular external and peer supervision. Family therapy was monitored to ensure the number and timing of sessions was as planned. With consent, sessions were video recorded to facilitate supervision. A random sample of videotapes (at least two per therapist) were independently rated by trained raters with clinical experience to measure adherence to the core elements of the manualised family therapy, using a structured rating scale^18^ (scores were 0–5 for adherence and 0–6 for competence; higher scores indicate greater adherence or competence).

Treatment as usual was offered to young people by local CAMHS teams and was unrestricted. We expected treatment as usual to be diverse and involve individual or family-orientated work, or both, delivered by a range of practitioners with different theoretical orientations. Clinicians who were involved with families in both groups could refer patients for additional specialist assessment and treatment as necessary. Further information about the therapy interventions is in the appendix.

Questionnaires for suicide ideation, quality of life, depression, mental health, family functioning, self-harm, emotional traits, health economics, and engagement with therapy were administered at baseline and 3, 6, 12, and 18 months after random group assignment, or different combinations of these timepoints (table 1). Questionnaires were completed by the young person, caregiver, or both. One questionnaire was also completed by the therapist.

**Table 1:** Questionnaire assessments

	**What is assessed**	**Completed by**	**Timepoint**
Beck Scale for Suicide Ideation	Suicidal ideation: intent and the severity of actual suicidal wishes and plans	Young person	Baseline, 12, and 18 months
Paediatric Quality of Life Enjoyment and Satisfaction questionnaire	Quality of life of young person: general	Young person	Baseline, 12, and 18 months
General Health Questionnaire-12	Quality of life of caregiver: mental health	Caregiver	Baseline, 12, and 18 months
Children's Depression Rating Scale—revised	Depression: severity of depressive syndrome	Young person	Baseline, 12, and 18 months
Strengths and Difficulties Questionnaire	Overall mental health and emotional and behavioural problems	Young person and caregiver	Baseline, 12, and 18 months
Hopelessness Scale for Children	Hopelessness: degree to which young people have negative expectancies of themselves and the future	Young person	Baseline, 12, and 18 months
McMaster Family Assessment Device	Family functioning	Young person and caregiver	Baseline, 12, and 18 months
Family Questionnaire	Family functioning: different ways in which families try to cope with everyday problems and expressed emotion	Young person and caregiver	Baseline, 3, and 6 months
Suicide Attempt Self-Injury Interview	Self-reported self-harm: factors involved in non-fatal suicide attempts and intentional self-injury, providing a timeline of self-harm episodes	Young person	Baseline, 12, and 18 months
Inventory of Callous Unemotional Traits	Callous, uncaring, and unemotional traits of young person	Young person and caregiver	Baseline
EQ-5D-3L	Health economics: health problems across dimensions, converted into health utilities	Young person	Baseline, 6, 12, and 18 months
Health Utilities Index 3	Health economics: quality of life health status classification system, converted into health utilities	Caregiver	Baseline, 6, 12, and 18 months
Health economics questionnaire	Trial-specific questionnaire for health economics	Young person and caregiver	Baseline, 3, 6, 12, and 18 months
System for Observing Family Therapy Alliances	Engagement with therapy	Young person, caregiver, and therapist	3 months

When it was not possible to arrange face-to-face follow-up interviews, and participants agreed, self-report questionnaires were posted to participants to complete. Postal questionnaires were sent at 3 and 6 months after random group assignment, preceded by a phone call from researchers.

All treatment data acquired after randomisation were provided by treating CAMHS clinicians, family therapists, clinical studies officers from local research networks, and researchers after participant follow-up was complete and masking unnecessary.

Hospital attendance data were obtained from accident and emergency and inpatient hospital episode statistics datasets from NHS Digital. These data were augmented by directed hospital record searches, undertaken by masked researchers as required throughout the trial.^19^

### Outcomes

The primary outcome was repetition of self-harm leading to hospital attendance in the 18 months after group assignment.

Secondary outcomes (at 12 and 18 months unless indicated) were repetition of self-harm leading to hospital attendance in the 12 months after group assignment; cost per self-harm event avoided because of family therapy (health economics analysis); characteristics (timing, number, severity, and dangerousness of method used) of all further self-harm episodes (those resulting in hospital attendance and all self-reported episodes); suicidal ideation; quality of life; depression; overall mental health and emotional and behavioural difficulties; hopelessness; family functioning; identification of moderating and mediating variables of adherence and engagement and benefit from treatment; therapeutic alliance to family therapy; and therapist adherence to the family therapy manual.

Non-serious adverse events were defined as attendance at accident and emergency and minor injury and walk-in centres, and re-referral to CAMHS, because these events were expected to occur within our study population. Serious adverse events were defined as deaths and hospital admissions. Adverse event data were obtained through researcher collection of data from CAMHS, Acute Trusts, and via hospital episode statistics from NHS Digital.

### Statistical analysis

We calculated that 832 participants (with 172 total events) were required to provide 90% power at a 5% significance level to detect the minimally important reduction in 18-month hospital admission for self-harm repetition—an absolute risk reduction of 10%, from 29% in the treatment as usual group^20^ to 19% in the family therapy group—using a log-rank test, providing a constant hazard ratio of 1·64, and assuming a loss to follow-up at 18 months of 10% or less.

Analyses described in a prespecified statistical analysis plan, approved by independent oversight committees, were done in the intention-to-treat population. All statistical testing used two-sided 5% significance levels and was done using SAS, version 9.4. Formal interim analysis of the primary endpoint, after 86 events, was reviewed by the Data Monitoring and Ethics Committee, who recommended recruitment of the full sample.

We analysed the primary outcome using Cox's proportional hazards, accounting for covariates (minimisation factors [sex, age, living arrangements, two *vs* three previous self-harm episodes, and index episode type] and NHS trust) to test for differences in the number of hospital attendances and times to attendance. Kaplan–Meier curves were used to show time to self-harm for each group. Participants without an event were censored at the time last known to be event free. We did a sensitivity analysis examining the effect of clustering (for therapists and NHS trusts) using multilevel survival frailty models,^21^ and the impact of unclassified hospital attendances (unknown whether because of self-harm or not).

We analysed secondary outcomes, including recurrent events analysis, on the basis of the Andersen-Gill^22^ method to analyse all hospital attendances because of self-harm. Repeated measures models (covariance pattern) adjusted for baseline score and covariates estimated treatment differences for questionnaire responses. Moderator variables (appendix) were assessed via interaction effects in the primary endpoint analysis, and those meeting the 5% significance level are reported. We did an exploratory complier average causal effect analysis, using instrumental variable probit regression, to model the causal effect of family therapy (regardless of initial treatment group assignment) on the primary outcome. Further mediator analysis methods and results are described in the appendix.

Participants with missing data for time-to-event outcomes (hospital attendance resulting from self-harm and primary, recurrent, and moderator analyses) were censored at baseline. Multiple imputation, assuming data were missing at random, was used to account for missing questionnaire data;^23^ complete case formed a sensitivity analysis.

A within-trial health economic evaluation compared outcomes and costs over 18 months of follow-up using trial data with quality-adjusted life-years (QALYs) and hospital attendance for a self-harm event avoided because of family therapy. Responses to the adolescent self-completed EQ-5D-3L questionnaire for measuring generic health status created a health profile of five digits,^24^ which was converted into utility by use of standard UK tariff values^25^ and multiplied by time spent in each state to generate QALYs. The choice of EQ-5D-3L was guided by a pilot study.^26^ Costs were based on resource usage incurred to NHS and social services while providing family therapy or treatment as usual, and incorporated hospital, primary, community, or social care service attendance, and medications reported by participants over the 18-month period (appendix). Resource usage was converted into costs using unit cost figures from the PSSRU Costs of Health and Social Care^27^ and Department of Health Reference Costs.^28^ Missing data were imputed using multiple imputations via chained equations as recommended for economic analyses alongside clinical trials.^29^ The cost of the intervention was calculated separately for the family therapy and treatment as usual groups, including any treatment details recorded (duration, number of therapists involved, type, attendance, telephone contact, and supervision meetings). April, 2014, to March, 2015, was used as the reference financial year. The discount rate was 3·5% as per NICE guidelines.^25^ Parameter uncertainty was addressed through probabilistic sensitivity analysis. Non-parametric bootstrapping generated simu-lations of mean costs and effects for each group.

A decision analysis model evaluated the cost-effectiveness of family therapy versus treatment as usual over a longer time period (5 years), using a Markov model with three health states: stop self-harm, repeat self-harm leading to hospital attendance, and death. Parameter uncertainty was addressed through probabilistic sensitivity analysis using MonteCarlo simulation (appendix).

We present outputs of the within-trial analysis and the decision analysis model as expected incremental cost-effectiveness ratios of family therapy versus treatment as usual using scatter plots on the cost-effectiveness plane and cost-effectiveness acceptability curves. Sensitivity analyses varied key parameters, including the number of therapists involved in family therapy and aggregated QALYs, for which the QALY gains for caregiver and young person were simply summed.^30^, ^31^ Caregivers' health-related quality of life was assessed using the Health Utilities Index version 3,^32^ and responses were converted into utility values.^33^ QALYs were calculated in the same manner as the young persons' QALYs. The trial is registered at the ISRCTN registry, number ISRCTN59793150.

### Role of the funding source

The funder of the study had no role in study design, data collection, data analysis, data interpretation, or writing of the report. The corresponding author had full access to all data in the study and had final responsibility for the decision to submit for publication.

## Results

**Figure 1:**
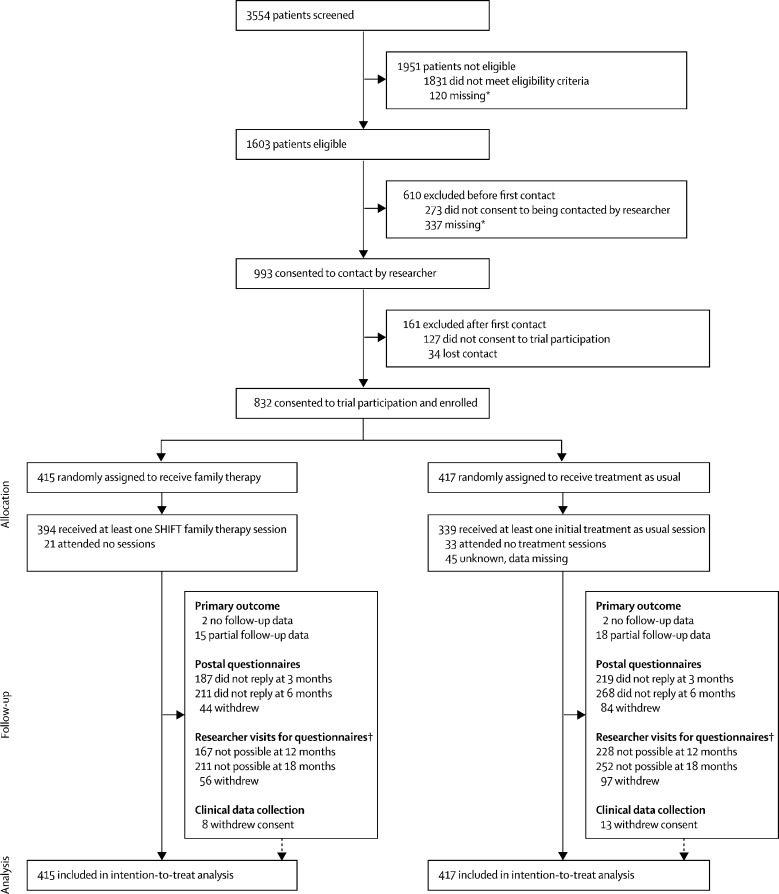
Trial profile A full list of reasons for dropouts at each stage of the study is provided in the appendix. SHIFT=Self-Harm Intervention: Family Therapy. *Data was not obtained for these people because clinical services did not fill in forms or contact was lost. †Reasons for loss to follow-up were unable to contact, contacted but unable to arrange visit, withdrawal from researcher visits, and visit arranged but cancelled or no one home.

Between Nov 23, 2009, and Dec 31, 2013, 3554 young people were screened and 1603 (45%) were deemed eligible. 993 (62%) of 1603 eligible young people consented to researcher contact, and 832 (52%) were then randomly assigned to receive family therapy (n=415) or treatment as usual (n=417; figure 1). All patients assigned to treatment were included in the analysis. One patient was younger than 18 years at the point of screening, but turned 18 just before random group assignment; this person was included in the intention-to-treat analysis.

Baseline characteristics between groups were similar at random group assignment (table 2; appendix). Mean age was 14·3 years (SD 1·4), and 737 (89%) of the 832 young people were female. 739 (89%) had self-harmed on at least three previous occasions; the most recent episode leading to CAMHS referral was self-injury for 594 (71%) patients and self-poisoning for 184 (22%) patients, with a further 54 (6%) patients being referred for both of these reasons. Suicide Attempt Self-Injury Interview (SASII) scores indicated that 516 (62%) participants met the criteria for non-suicidal self-injury. 830 (>99%) of the 832 participants were living with their parents or guardians rather than in foster care, and 784 (94%) were in full-time education.

**Table 2:** Young person baseline characteristics

		**Family therapy (n=415)**	**Treatment as usual (n=417)**	**Total (n=832)**
Sex				
	Female	368 (89%)	369 (88%)	737 (89%)
	Male	47 (11%)	48 (12%)	95 (11%)
Age, years				
	11–14	220 (53%)	221 (53%)	441 (53%)
	15–17	195 (47%)	195 (47%)	390 (47%)
	18	0	1 (<1%)	1 (<1%)
Number of known self-harm episodes				
	Two	46 (11%)	47 (11%)	93 (11%)
	At least three	369 (89%)	370 (89%)	739 (89%)
Type of most recent episode				
	Self-poisoning	93 (22%)	91 (22%)	184 (22%)
	Self-injury	297 (72%)	297 (71%)	594 (71%)
	Combined	25 (6%)	29 (7%)	54 (6%)
SASII interviewer-rated behaviour				
	Suicide attempt	148 (36%)	165 (40%)	313 (38%)
	Non-suicidal self-injury	265 (64%)	251 (60%)	516 (62%)
	Non-intentional self-injury (victim precipitated, did not act)*	2 (<1%)	1 (<1%)	3 (<1%)
SASII interviewer rated intent to die (at least some intent to die)		197 (47%)	215 (52%)	412 (50%)
Other baseline criteria				
	Referred to CAMHS via hospital	156 (38%)	148 (35%)	304 (37%)
	Previous CAMHS involvement reported	136 (33%)	108 (26%)	244 (29%)
	Young people reported to be taking a prescribed psychotropic†	17 (4%)	24 (6%)	41 (5%)
	Physical health problem or disability reported	110 (27%)	108 (26%)	218 (26%)
	Parental abuse reported by young person‡	89 (21%)	109 (26%)	198 (24%)
	Any marked physical abuse reported by the young person (parental or other)	80 (19%)	98 (24%)	178 (21%)
	Any sexual abuse reported by the young person	75 (18%)	63 (15%)	138 (17%)
	Young person in full-time education	398 (96%)	386 (93%)	784 (94%)

Data are n (%). SASII=Suicide Attempt Self-Injury Interview. CAMHS=Child and Adolescent Mental Health Services.

* Included as eligible because all three young people had other previous self-harm confirmed in the SASII timeline, and these three events were included, because in the judgement of the research team, the young people were in the process of self-harming as described in the eligibility criteria but the act was interrupted either by the young person or another person.

† Includes psychotropic medications for attention deficit hyperactivity disorder (six people), anti-anxiety (one), antipsychotic (two), and antidepressant (28) medications, and sedatives or sleep medications (five).

‡ Parental, marked physical, and sexual abuse were each reported as separate events.

Baseline data showed that a higher proportion of participants had experienced marked difficulties in our cohort than is typical for young people referred to CAMHS, indicating a higher-risk population,^17^ as would be expected for young people who had self-harmed at least twice. 528 (63%) of the 832 participants were direct community referrals to CAMHS and the remaining 304 (37%) were recruited after an accident and emergency attendance resulting from self-harm (table 2). Additional baseline data from caregiver and young person questionnaires are in the appendix.

At the 18-month follow-up, 21 (3%) of the 832 participants had withdrawn consent for clinical data collection (eight in the family therapy group and 13 in the treatment as usual group; figure 1). Most participants were successfully linked to hospital episode statistics data based on participant identifiers. Full primary outcome data were available for 795 (96%) participants, partial data were available for 33 (4%), and no data were available for four (<1%).

Substantial loss to follow-up occurred for participant-reported secondary outcomes. At 12 months, researcher follow-up was possible for 248 (60%) participants in the family therapy group and 189 (45%) in the treatment as usual group. At 18 months, 204 (49%) of 415 participants in family therapy and 165 (40%) of 417 in the treatment as usual group were followed up. Overall at least one follow-up was completed for 498 (60%) of 832 participants. More losses to follow-up occurred in the treatment as usual group than in the family therapy group (figure 1); participants lost to follow-up had less favourable baseline characteristics in terms of their scores on the Strengths and Difficulties Questionnaire (SDQ), McMaster Family Assessment Device (FAD), Global Health Questionnaire-12 (GHQ-12), and Inventory of Callous-Unemotional Traits (ICU), and were more likely to have been referred into CAMHS from hospital. Results indicated that more participants with less favourable characteristics were lost to follow-up in the treatment as usual group than in the family therapy group, based on SDQ, FAD, and ICU scores (data not shown).

In the family therapy group, 21 (5%) of 415 young people attended no family therapy sessions and 394 (95%) attended at least one family therapy session; in the treatment as usual group, 33 (8%) of 417 attended no sessions of treatment as usual, 339 (81%) attended at least one session, and data were missing for 45 (11%) people. A median of six family therapy sessions were attended (IQR 3·0–9·0; range 0–21), lasting a median of 5·1 months. The pattern was more varied in the treat-ment as usual group: median of five sessions attended (IQR 2·0–12·5; range 0–163), over 4·4 months.

The treatment given to patients in the treatment as usual group varied considerably, with the most common type being supportive therapy or counselling. The other most used types of therapy included cognitive behavioural therapy, family work (discussion meetings with families without formal family therapy), and formal systemic family therapy (appendix). About 20% of sessions were for assessment or review rather than therapy (appendix).

Analysis of 52 family therapy video recordings (23 first sessions, 29 later sessions, involving 26 therapists, in patients in the family therapy group) showed good adherence of therapists to the manual (mean score 4·6 [SD 0·72]) and good competence (4·4 [1·03]) in conducting the sessions.

**Figure 2:**
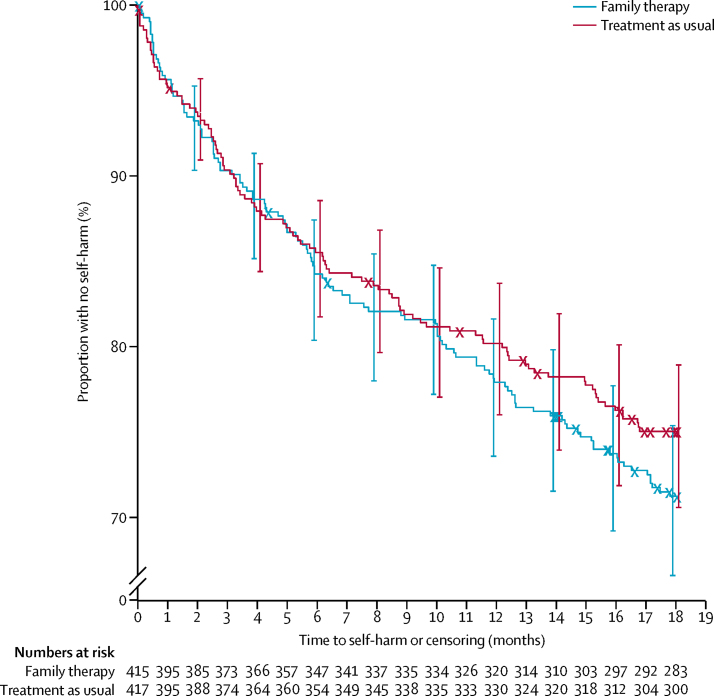
Kaplan-Meier plot of time to self-harm Bars show 95% CI.

At the 18-month follow-up, 221 (27%) of 832 young people had attended hospital after repeated self-harm; 118 (28%) of 415 in the family therapy group and 103 (25%) of 417 in the treatment as usual group (figure 2); the hazard ratio for family therapy versus treatment as usual was 1·14 (95% CI 0·87–1·49; p=0·33; table 3). Sensitivity and exploratory (complier average causal effect) analyses confirmed primary analysis results (table 3; appendix). Repeated self-harm was less common in male participants and those who were 15 years or older. Proportions of patients who repeated self-harm varied substantially across study centres, and were higher in the subgroups of participants referred to CAMHS via hospital (*vs* those referred via the community) and those with an index episode combining self-injury and poisoning (*vs* either method alone). The method used for the primary self-harm outcome event was often different from that for the index event, and more than half of participants who repeated by self-poisoning had self-injured at their index episode.

**Table 3:** Results of primary and secondary outcomes for the risk of repeat self-harm in the treatment groups and covariates

		**Primary outcome**								Secondary outcomes*			
		Primary analysis: Cox proportional hazards for first event		Sensitivity analysis: frailty model, clustering by therapist		Sensitivity analysis: frailty model, clustering by NHS trust		Sensitivity analysis: including unclassified hospital attendances†		Cox proportional hazards for first event (within 12 months)		Recurrent events	
		Hazard ratio (95% CI)	p value	Hazard ratio (95% CI)	p value	Hazard ratio (95% CI)	p value	Hazard ratio (95% CI)	p value	Hazard ratio (95% CI)	p value	Hazard ratio (95% CI)	p value
**Fixed effects**													
Treatment: family therapy (*vs* treatment as usual)		1·14 (0·87–1·49)	0·33	1·09 (0·81–1·46)	0·50	1·14 (0·87–1·48)	0·33	1·15 (0·89–1·49)	0·27	1·09 (0·81–1·48)	0·56	1·05 (0·76–1·44)	0·78
Sex: female (*vs* male)		1·60 (0·98–2·61)	0·059	1·61 (0·98–2·63)	0·057	1·54 (0·94–2·50)	0·084	1·65 (1·03–2·65)	0·039	1·60 (0·92–2·79)	0·094	1·27 (0·77–2·10)	0·34
Age group: 15–17 years (*vs* 11–14 years)		0·70 (0·53–0·92)	0·011	0·69 (0·52–0·91)	0·0095	0·70 (0·53–0·93)	0·012	0·75 (0·58–0·98)	0·038	0·72 (0·53–0·99)	0·043	0·67 (0·50–0·92)	0·012
Previous self-harm episodes: at least three (*vs* two)		1·22 (0·78–1·92)	0·39	1·21 (0·77–1·91)	0·41	1·22 (0·78–1·92)	0·38	1·20 (0·78–1·85)	0·41	1·31 (0·77–2·22)	0·32	1·52 (0·92–2·49)	0·10
Type of index episode		..	0·033	..	0·035	..	0·023	..	0·020	..	0·071	..	0·064
	Combined (*vs* injury)	1·83 (1·14–2·96)	..	1·83 (1·13–2·98)	..	1·85 (1·15–2·98)	..	1·90 (1·20–3·02)	..	1·80 (1·05–3·09)	..	1·20 (0·66–2·18)	..
	Poisoning (*vs* injury)	1·03 (0·69–1·54)	..	1·02 (0·68–1·53)	..	1·00 (0·67–1·49)	..	1·09 (0·74–1·60)	..	1·00 (0·63–1·57)	..	0·72 (0·45–1·16)	..
Referred via hospital: yes (*vs* community)		1·31 (0·93–1·86)	0·12	1·33 (0·93–1·88)	0·11	1·39 (0·99–1·95)	0·060	1·24 (0·88–1·74)	0·21	1·27 (0·86–1·88)	0·23	1·98 (1·18–3·32)	0·0096
NHS trust		..	0·094	..	0·15	..	..	..	0·077	..	0·14	..	0·049
**Random effects**													
Main therapist		..	..	..	0·37	..	..	..	..	..	..	..	..
NHS trust		..	..	..	..	..	0·065	..	..	..	..	..	..

‡p values are for type of index episode (injury, poisoning, or combined).

* Further secondary analysis using adjusted probit regression found similar estimates for the intention-to-treat, as treated, and complier average causal effect analysis. There was a similar effect of family therapy receipt in the complier average causal effect analysis (parameter estimate 0·12 [SE 0·13], p=0·34) compared with the standard intention-to-treat estimate of the allocation of family therapy (0·11 [SE 0·10], p=0·24), and the as treated estimate (0·10 [SE 0·10], p=0·31); with no significant differences detected between trial groups, or receipt of family therapy.

† 47 unclassified attendances in 41 participants were classed as being related to self-harm, thus contributing new primary outcome events for 18 participants, and earlier primary outcome events for nine participants.

Neither the proportion of people who attended hospital after repeated self-harm within 12 months nor the number of recurrent events were significantly different between the two groups (table 3).

Young people's questionnaire outcomes for depression (Children's Depression Rating Scale—revised), quality of life (Paediatric Quality of Life Enjoyment and Satisfaction questionnaire), hopelessness (Hopelessness Scale for Children), family functioning (FAD), caregiver mental health (GHQ-12), and expressed emotion (the Family Questionnaire) were not significantly different between the two groups (table 1; appendix). Family therapy participants reported significantly better behaviour outcomes on subscales of the SDQ for the young person and caregiver, young person suicidal ideation (Beck Scale for Suicide Ideation), and caregiver family functioning (FAD; appendix) than the treatment as usual group. Because of substantial and differential loss to follow-up, between-group differences should be interpreted with caution. However, complete-case sensitivity analysis found similar results to those reported by use of multiple imputation, with no change to conclusions.

Self-harm was self-reported on the SASII for 349 (73%) of 478 participants during the 12–18 months follow-up for whom data were available; 202 (75%) of 268 in family therapy and 147 (70%) of 210 in treatment as usual.

The number of participants referred to other services (including inpatient units) and safety outcomes (re-referrals to CAMHS, accident and emergency attendances, and hospital admissions for any reason; table 4) were similar for both groups. 1036 adverse events were reported for 443 (53%) of the 832 participants (226 [54%] of 415 in the family therapy and 217 [52%] of 417 in the treatment as usual group), with a mean of 1·2 events per participant (SD 2·1). 598 serious adverse events were reported for 297 (36%) participants (156 [38%] of 415 in the family therapy and 141 [34%] of 417 in the treatment as usual group). No deaths were reported (table 4).

**Table 4:** Adverse events

		**Family therapy (n=415)**	**Treatment as usual (n=417)**	**Total (n=832)**
**Non-serious adverse events**				
Number of participants with one or more adverse event		226 (54%)	217 (52%)	443 (53%)
	Accident and emergency attendance*	189 (46%)	176 (42%)	365 (44%)
	Minor injury or walk-in centre attendance*	33 (8%)	40 (10%)	73 (9%)
	Re-referral to CAMHS	52 (13%)	56 (13%)	108 (13%)
Number of adverse events reported		512	524	1036
	Accident and emergency attendance	409	372	781
	Minor injury or walk-in centre attendance	45	89	134
	Re-referral to CAMHS	58	63	121
Mean (SD) adverse events per participant		1·2 (2·0)	1·3 (2·3)	1·2 (2·1)
Median (IQR) adverse events per participant		1·0 (0·0–2·0)	1·0 (0·0–2·0)	1·0 (0·0–2·0)
**Serious adverse events**†				
Number of participants with one or more serious adverse event		156 (38%)	141 (34%)	297 (36%)
Number of serious adverse events reported		275	323	598
Mean (SD) serious adverse events per participant		0·7 (1·3)	0·8 (1·9)	0·7 (1·6)
Median (IQR) serious adverse events per participant		0·0 (0·0–1·0)	0·0 (0·0–1·0)	0·0 (0·0–1·0)

* Accident and emergency attendances, minor injury or walk-in centre attendances, and hospital admissions were for any mental health or non-mental health reason, and so include self-harm.

† All serious adverse events were hospital admissions; no deaths were reported.

Significant moderation of the primary outcome was detected for the unemotional subscale of the young person-reported ICU, and the affective involvement subscale of the caregiver-reported family functioning measure (FAD). No moderation was detected for other baseline factors (appendix).

**Figure 3:**
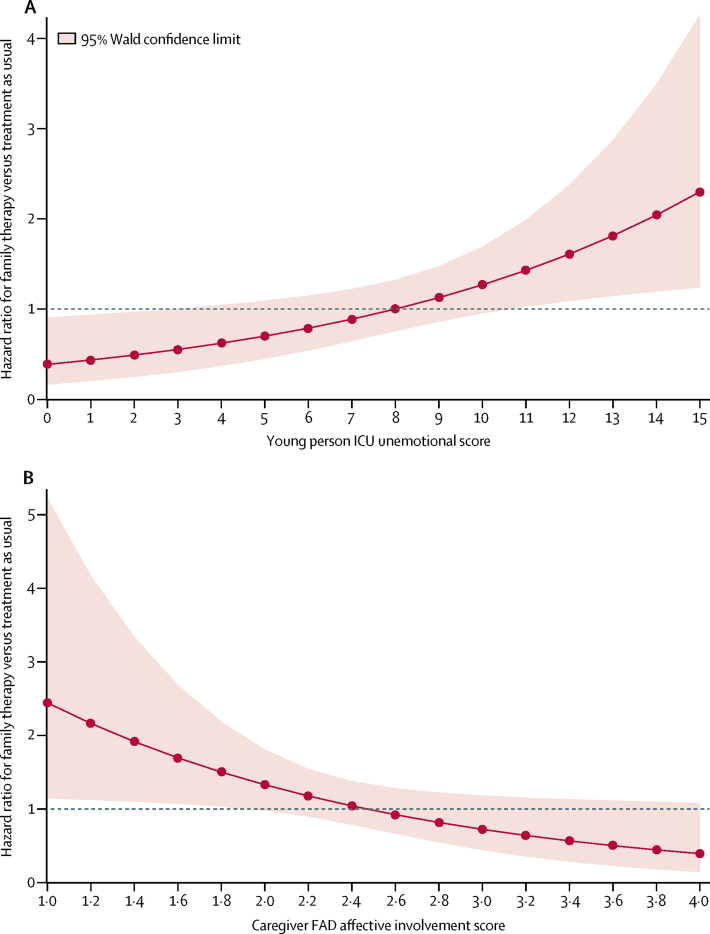
Moderator analysis: hazard ratio for risk of hospital attendance due to repeat self-harm (A) Baseline young-person ICU unemotional subscale score (range 0–15). Higher scores indicate more unemotional traits. (B) Baseline caregiver FAD affective involvement subscale score (range 1–4). Higher scores indicate poorer family functioning. FAD=Family Assessment Device. ICU=Inventory of Callous Unemotional Traits.

In young people who reported difficulty talking about feelings on the ICU, those in the family therapy group had an increased risk of hospital attendance for repeat self-harm versus those in the treatment as usual group; whereas in young people who reported that they could discuss feelings easily, those in the family therapy group had a decreased risk versus those in the treatment as usual group (p=0·010; figure 3A). This moderation was such that as the young person reported greater difficulty in talking about feelings (1 point increase on the ICU, equivalent to worse or more severe trait), the risk of self-harm increased in the family therapy group (HR 1·05 [95% CI 0·98–1·12]) and decreased in the treatment as usual group (HR 0·93 [0·88–0·99]). Conversely, as the young person reported less difficulty talking about feelings (1 point decrease on the ICU), the risk of self-harm decreased in the family therapy group (HR 0·95 [95% CI 0·89–1·02]) and increased in the treatment as usual group (HR 1·08 [1·01–1·14]).

In young people whose caregivers reported healthier affective involvement (degree to which family members are involved and interested in one another) on the FAD, those in the family therapy group had a higher risk of self-harm than those in the treatment as usual group; whereas in young people with poorer affective involvement, those in family therapy had a decreased risk versus those in treatment as usual (p=0·034; figure 3B). This moderation was such that as the caregiver reported more affective involvement problems (1 point increase on the FAD, equivalent to worse or more severe trait), the risk of self-harm decreased in the family therapy group (HR 0·89 [95% CI 0·59–1·34]) and increased in the treatment as usual group (HR 1·63 [95% CI 1·11–2·39]; appendix). Conversely, as the caregiver reported fewer affective involvement problems (1 point decrease on the FAD), the risk of self-harm increased in the family therapy group (HR 1·12 [95% CI 0·75–1·69]) and decreased in the treatment as usual group (HR 0·61 [95% CI 0·42–0·90]). Cost-effectiveness analyses showed an increase in the mean EQ-5D-3L score in both groups at the 18-month follow-up compared with baseline. Participants in the family therapy group incurred costs of GBP £1266 (95% CI 736 to 1796; p<0·0001), higher than those in the treatment as usual group, and gained 0·034 (95% CI −0·004 to 0·065; p=0·13) more quality-adjusted life-years (QALYs), equivalent to 12·4 more days of perfect health (table 5). The incremental cost-effectiveness ratio (ICER) of £36 812 per QALY was higher than the recommended threshold range specified for NICE decision making in England and Wales (which is a gain of £20 000–30 000 per QALY gained),^25^ showing that family therapy was probably not cost-effective. As the difference in QALY gains between family therapy and treatment as usual was marginal at 18 months and absent in the complete case analysis, decision analysis modelling was not required. Family therapy was less cost-effective than treatment as usual, with an estimated 0·033 more self-harm events (95% CI −0·130 to 0·197; p=0·98), and a cost increase of £1253 (95% CI 725 to 1780; p<0·0001). In all sensitivity analyses of intervention costs, the ICER remained greater than NICE's recommended threshold. However, combining young people's and caregivers' QALY gains, family therapy incurred higher costs than treatment as usual but better health outcomes (table 5). The corresponding ICER was £20 808 per QALY with a 41% probability to be cost-effective at £20 000 per QALY (64% probability at £30 000 per QALY).

**Table 5:** Results of cost-effectiveness analysis at 18 months

	**Estimated incremental cost**	**p value**	**Estimated incremental QALYs**	**p value**	**ICER, £ per QALY**
**Primary analysis**					
QALYs	£1266 (736 to 1796)	<0·0001	0·034 (−0·004 to 0·065)	0·13	£36 812
Secondary analysis					
Hospital attendance for repeated self-harm event	£1253 (725 to 1780)	<0·0001	0·033 (−0·130 to 0·197)*	0·98	Family therapy less effective and more costly than treatment as usual (family therapy is a dominated option)†
**Sensitivity analyses**					
Bootstrapped average (10 000 replications)‡	£1255 (1149 to 1260)	<0·0001	0·034 (0·034 to 0·034)	0·03	£36 706
Assumption of only one therapist involved in each session in the family therapy group‡	£1380 (748 to 2013)	<0·0001	0·034 (−0·004 to 0·065)	0·13	£40 130
Assumption of average number of therapists involved in each treatment session in the family therapy group‡	£1546 (910 to 2183)	<0·0001	0·034 (−0·004 to 0·064)	0·14	£44 956
Adjustment for baseline EQ-5D-3L differences‡	£1266 (736 to 1796)	<0·0001	0·039 (0·035 to 0·042)	0·03	£32 852
Complete case	£1135 (267 to 2538)	<0·0001	−0·003 (−0·086 to 0·080)	0·91	Family therapy less effective and more costly than treatment as usual (family therapy is a dominated option)
Including caregivers' QALYs‡	£1207 (662 to 1752)	<0·0001	0·058 (0·002 to 0·114)	0·04	£20 808

All results are estimates for family therapy versus treatment as usual (95% CI). QALY=quality-adjusted life-year. ICER=incremental cost-effectiveness ratio.

* Incremental number of self-harm events estimate (95% CI).

† ICER, £ per self-harm event.

‡ With multiple imputation.

## Discussion

In young people who had recently repeatedly self-harmed, we found no clinical or cost benefits for family therapy over treatment as usual in terms of hospital attendance for subsequent repetition of self-harm. For interventions in which the whole family was the subject of the assessment, and more than one person is involved in treatment, methods to assess benefits (and harms) beyond the individual should be considered.^30^ Our finding that family therapy is cost-effective when considering combined benefits to the young person and caregiver is therefore salient, although it assumes QALYs can be aggregated across individuals as a simple sum. This addition has been done in previous studies of child health,^34^ but is not part of the NICE reference case and assumes interdependence between utilities (the health state) of the adolescent and caregiver.

Our study sample had baseline levels of difficulty at least as severe as the average CAMHS referral.^17^ The proportion of female participants recruited and the mix of referrals from hospital and community sources are similar to those seen in other studies.^4^ The treatments given in the treatment as usual group were highly varied, which can be expected from a pragmatic trial, but broadly similar to CAMHS practice in the UK with a mixture of supportive or individual counselling, cognitive behavioural therapy, and family work;^17^ however, the proportion of patients who received formal family therapy for self-harm (87 [21%] of 417) was much higher than we were expecting. Those in the treatment as usual group who received a version of family therapy did not, however, have significantly better primary outcome results than either those in the family therapy group or those in treatment as usual who only received other interventions.

Although we report almost complete data for the primary outcome, loss to follow-up resulted in partial data from 478 (57%) of 832 participants for self-reported self-harm at 18 months. Strenuous efforts were made by the research team to track participants via clinic and general practitioner records, and by use of letters, emails, telephone calls, and text messages. More frequent follow-up might have maintained engagement with participants and improved follow-up but would have substantially added to the cost of the project and was not feasible while retaining an 18-month primary outcome. From the self-report data, many self-harm events did not lead to hospital attendance (the primary outcome), with 349 (73%) of 478 self-reporting self-harm; however, the self-reported results were similar between groups, and in line with the primary outcome finding that was based on hospital records. For other secondary outcomes, young people and their caregivers in the family therapy group reported significantly better outcomes on several elements of general emotional and behavioural difficulties (SDQ), suggesting family therapy had a significant positive effect on general mental health, even if this did not translate into reduced repetition of self-harm. Self-reported suicide ideation scores were lower in the family therapy group than in the treatment as usual group at 12 months but not at 18 months, consistent with other family therapy research.^35^ It might be argued that reducing suicidal ideation sooner is a potentially important clinical benefit. Furthermore, those lost to follow-up in the treatment as usual group seemed to have had less favourable baseline characteristics (eg, depression), meaning that the participants in this group who returned data might have been a less impaired group than those who returned data in the family therapy group. This factor makes comparison between the groups unreliable and might mask any advantages gained by the family therapy group.

In our trial, families who reported poorer family functioning scores in relation to affective involvement benefitted more from family therapy (*vs* treatment as usual) than those with better scores; whereas previous studies have suggested that the families with good functioning benefit most from family therapy.^36^ The finding that adolescents who find communication about feelings difficult might do less well in a family intervention, where such expression is encouraged, is in line with the literature. These two findings together suggest an association between adolescent and family functioning, and ability to talk about feelings, that is more complex than previously reported. Harrington and colleagues' study^37^ of a home-based intervention for self-harm found moderation by depression, with family therapy reducing suicidal ideation in the absence of depression in the young person.^37^ Families with high levels of criticism tend to drop out of family therapy for eating disorders and do worse in conjoint family therapy than when parents and adolescents are seen in parallel.^38^

Our study differs from other trials of interventions to reduce self-harm in its substantially larger sample size, long follow-up, and use of an objectively measured primary outcome (hospital attendance) that traced 96% of participants. For example, three other studies have reported positive outcomes for self-harm reduction following dialectical behaviour therapy (n=77),^39^ mentalisation-based treatment (n=80),^40^ and family psychoeducation (n=48),^41^ but all recruited far fewer participants than our study, and their primary outcomes used retrospective self-report questionnaires and interview measures—with potential for reporting biases. The most rigorous other published study, the ASSIST trial of routine care plus group therapy,^42^ assigned 366 young people and used masked-researcher-rated structured interview techniques to elicit the frequency of self-harm events, with good follow-up attendance. ASSIST also found no significant clinical or cost benefits for its primary outcome of frequency of repetition of self-harm.

Most young people in our trial (594 [71%] of 832) had self-injured for their index episode rather than self-poisoned (184 [22%]), differing markedly from the 17% in a monitoring study in three English cities of 5200 consecutive hospital attendances for self-harm in people younger than 18 years;^4^ the discrepancy is explained by the high proportion of trial participants recruited from community referrals in our study. Participants in our trial must have self-harmed at least twice, complicating comparison with other studies because previous self-harm is not always reported consistently. SASII ratings suggested that 516 (62%) of 832 patients' index events would be classified as non-suicidal self-injury but 412 (50%) of participants were rated by the researcher as having some intent to die; we deem the UK definition of self-harm, which disregards motive, to be justified because of ambiguity and difficulty interpreting statements of intent. The fact that the method used for the primary self-harm outcome event was often different from that for the index event (more than half of participants who self-poisoned at repetition had self-injured at their index episode) further challenges any assumption of non-suicidality in young people who self-injure.

The SHIFT version of family therapy placed great emphasis on families discussing self-harm and formulating strategies to deal with subsequent events—often involving the seeking of help from external agencies. Therefore, the intervention itself might have differentially increased presentation to hospital for self-harm and as a result, confounded results. However, the large proportion of missing self-reported data makes it difficult to test this hypothesis in this trial. The pragmatic design of this trial also conferred a potential advantage on treatment as usual because experienced CAMHS clinicians in the treatment as usual group had flexibility in selecting treatments on the basis of clinical assessment and discussions with the young person and family about preferred treatment strategies.

The substantial loss to follow-up markedly limited the interpretation of secondary participant-reported questionnaire outcomes and health economic analyses. The large proportion of missing data, imputed through multiple imputation, increases the variability in secondary outcomes, limiting inferences where non-significant differences were detected. Analysis relies on the missing at random assumption, with missingness fully explained through observed data; covariates and treatment were therefore included in our imputation model to support this as a plausible assumption.

The low intensity of treatment given in our family therapy intervention could have reduced the chance of finding a positive effect of family therapy. The average number of sessions in UK CAMHS clinics^17^ is low relative to those in some studies of psychological inteventions, but we received a strong clinical message from collaborating sites that participants who had self-harmed were difficult to engage in treatment and would not adhere to a longer and more intensive intervention than that which was delivered in our study. Because this was a pragmatic trial, we designed an intervention that would be broadly equivalent in the number of sessions to those in the treatment as usual and likely, if successful, to be funded by the UK health-care system. No a priori evidence exists for an advantage of longer duration of therapy. In practice, participants in the family therapy and treatment as usual groups did receive broadly similar numbers of sessions. A more intensive intervention might have been more successful but might also have led to concerns that positive findings were as a result of the intensity of the intervention rather than the intervention itself. The same arguments might apply to the ASSIST trial in which the mean number of sessions attended for the core intervention was 10·2 (*vs* a median of six sessions in our study).^42^

The SHIFT trial shows that this version of family therapy did not confer extra benefits in reducing the risk of hospital attendance after further self-harm. The trial participants were a high-risk group, having self-harmed at least twice, and comparisons cannot be drawn with those presenting for the first time after self-harm. Some evidence supports the increased effectiveness of family therapy versus treatment as usual in reducing self-harm in cases in which caregivers report poor family functioning, or young people report ease in discussing emotions. Conversely, when young people report difficulty in expressing emotion, or families report healthy functioning, other interventions or modifications of family therapy might be indicated.

The findings might not be generalisable to the subset of adolescents who present to hospital in the UK after a first episode of self-harm. However, this trial does confirm that young people are likely to switch methods in subsequent episodes of self-harm and that the method of self-harm might not be a useful indicator of suicidal intent or risk. The trial provides some evidence that family therapy has a positive effect on general emotional and behavioural problems and might reduce suicidal ideation faster than treatment as usual, albeit based on self-report measures with substantial amounts of missing data. Young people who self-harm form a varied and heterogeneous group with self-harm likely to be the final common pathway for a wide range of predicaments. Future research needs to explore the characteristics of specific subgroups within the self-harming population. Possible candidate groups arising from this research would be families who self-report poorer family functioning, young people who are more unemotional, and those referred to CAMHS directly from the hospital rather than from the community. Further analysis of the effect modifiers in SHIFT might have a useful explanatory or theoretical function and help develop effective interventions. Longer follow-up studies are needed and the possibility that family therapy might have had benefits beyond that of the participant deserves further exploration, including how health economic benefits might be aggregated for family members.

For the **SHIFT protocol** see http://www.trialsjournal.com/content/16/1/501
