# Supplementary material for: Effectiveness of systemic family therapy versus treatment as usual for young people after self-harm: a pragmatic, phase 3, multicentre, randomised controlled trial
Source: Lancet Psychiatry. 2018 Mar;5(3):203–16. doi: 10.1016/S2215-0366(18)30058-0 (PMC5835764; doi:10.1016/S2215-0366(18)30058-0)
Supplement: Supplementary appendix [file mmc1.pdf]

THE LANCET
Psychiatry

Supplementary appendix

This appendix formed part of the original submission and has been peer reviewed. We post it as supplied by the authors.

Supplement to: Cottrell DJ, Wright-Hughes A, Collinson M, et al. Effectiveness of systemic family therapy versus treatment as usual for young people after self-harm: a pragmatic, phase 3, multicentre, randomised controlled trial. *Lancet Psychiatry* 2018; published online Feb 12. http://dx.doi.org/10.1016/S2215-0366(18)30058-0.

Effectiveness and cost-effectiveness of systemic family therapy compared with treatment as usual for young people after self-harm: a pragmatic randomised controlled trial. Cottrell et al, 2018, Lancet Psychiatry

Appendix

Table of Contents

1. Inclusion and exclusion criteria and their rationale: Table 1 2
2. Family therapy and TAU interventions: Table 2 4
3. Full young person and caregiver scores at baseline: Tables 3 & 4 7
4. Full young person and caregiver scores on secondary outcomes at 12 and 18 months: Tables 5 & 6 11
5. Predictive and process measures: Figure 1; Tables 7 & 8 15
6. Comparison of baseline scores with population data 7. 19
7. Health Economics: Intervention Costs: Tables 9 & 10 20
8. Health Economics: Monte Carlo simulation, model parameters: Figure 2 and Table 11 23
9. References 26

1. Inclusion and exclusion criteria and their rationale
This was a pragmatic trial involving young people who could be at significant risk of further self-harm. In deciding on inclusion and exclusion criteria we had to balance the need to be inclusive, in order to be as representative as we could of real-world practice, against the very real anxieties of local clinical services about randomising to interventions, young people at risk of further self-harm.

Our funder specified that the trial must include young people, aged 11-17, who had self-harmed at least once before this index presentation. In common with UK, European and Australasian practice, we defined self-harm as any form of intentional non-fatal self-poisoning or self-injury (including cutting, taking excess medication, hanging, self-strangulation, jumping from height, and running into traffic), regardless of suicidal intent; this includes the US definitions of non-suicidal self-injury (NSSI) and suicidal behaviour.

In addition, we specified that:

- The self-harm had to have led to referral to a CAMHS team (we did not have the resources to identify participants in the community);
- Participants were living with a primary caregiver who was willing to take part in therapy (as we were evaluating a family-based intervention we needed to know that there was at least one other family member/ caregiver willing to be involved);
- If the index episode was due solely to alcohol or recreational drugs, participants had explicitly stated that they intended self-harm;
- It was the intention of the CAMHS team to offer their usual out-patient treatment – see exclusion criteria.

In considering exclusion criteria our concern was primarily to ensure participant safety and the integrity of the research process. Our exclusion criteria were:

- Serious suicide risk making outpatient treatment unsafe in the opinion of CAMHS clinicians. (Most young people who self-harm in the UK are assessed quickly by a CAMHS clinician and offered out-patient follow-up treatment. In a few cases where suicide risk is judged to be very high, local clinicians may make alternative treatment arrangements: in-patient admission, intensive outreach services etc. In such cases randomisation to TAU would not be possible as clinical judgement deemed other treatments urgently necessary);
- The need for specialist CAMHS services (most UK CAMHS teams have specialist clinics for a few serious specific conditions, for example, early intervention in psychosis, eating disorders. In such cases randomisation to TAU would also not be possible as clinical judgement deemed other treatments necessary);
- Current pregnancy (the need to attend ante and post-natal care was likely to interfere with the usual delivery of TAU or FT);
- Current CAMHS treatment (it was considered unethical to potentially disrupt already ongoing treatment to randomise to a new treatment);
- Sibling in trial or receiving family therapy (it was considered unethical to potentially disrupt already ongoing treatment to randomise to a new treatment);
- Living in a children's home or short-term foster placement where changes of placement were likely, and it was therefore unlikely that a stable caregiver presence at FT would be possible). Participants were eligible if living in longer term foster care as long as other criteria were met;
- Moderate to severe learning disability (if lacking capacity to comply with trial requirements of consent or of involvement in treatment);
- Involved in conflicting research (it was considered unethical to involve a young person in a second possibly conflicting research project)
- Insufficient proficiency in English to provide self-reported data (interpreters were available for therapy sessions in both arms of the trial. This exclusion criterion related only to inability to complete self-report data).

See Consort Diagram, Figure 1 in main paper. Additional data on reasons for exclusion are presented in Appendix Table 1.

Table 1: Recruitment data, reasons for ineligibility, no contact and non-consent

	N	(%)
**Total Screened**	**3554**	
**Reasons for ineligibility**		
Engaged in <1 prior self-harm episode	821	(44.8)
Already being treated in CAMHS	461	(25.2)
Not intended to offer CAMHS follow-up for self-harm	397	(21.7)
Does not live with primary caregiver	323	(17.6)
Recent self-harm not a key feature of presentation	300	(16.4)
Due to alcohol / drugs, specific intent to self-harm not established	250	(13.7)
In a children's home or short-term foster placement	183	(10.0)
Would not ordinarily be treated in CAMHS	169	(9.2)
Currently undergoing child protection investigation	143	(7.8)
At serious risk of suicide	129	(7.0)
Insufficient proficiency in English	58	(3.2)
Learning disability / lacks Capacity	49	(2.7)
Not aged 11-17	42	(2.3)
Involved in other research project	19	(1.0)
Pregnant	9	(0.5)
Sibling in SHIFT/CAMHS family therapy	7	(0.4)
Unknown	36	(2.0)
Total ineligible	1831	(51.5)
Missing screening data	120	(3.4)
**Total Eligible of total screened**	**1603/3554**	**(45.1)**
**Reasons for lack of contact with researcher**		
Refused consent	160	(58.6)
Did not attend first follow-up appointment	85	(31.1)
Not approached by CAMHS clinician	18	(6.6)
Unknown	10	(3.7)
Of those who refused:		
Young person	67	(41.9)
Parent / caregiver	22	(13.8)
Both	64	(40.0)
Missing	7	(4.4)
Total with no contact	273	(17.0)
Missing contact information	337	(21.0)
**Total Consented to Researcher contact of total eligible**	**993/1603**	**(61.9)**
Reasons for non-consent		
Refused	100	(78.7)
Unknown	16	(12.6)
Could not contact	11	(8.7)
Of those who refused:		
Young person	55	(55.0)
Parent / caregiver	19	(19.0)
Both	25	(25.0)
Missing	1	(1.0)
Total who did not consent	127	(12.8)
Missing consent information	34	(3.4)
**Total randomised of total consenting to researcher contact**	**832/993**	**(83.8)**

2. Family therapy and TAU interventions
Therapeutic interventions for both arms of the trial were delivered within CAMHS, and all participants were treated within their local service. Family therapists were formally linked with specific CAMHS teams to ensure lines of clinical responsibility were clear. Appendix Table 2 sets out the broad therapeutic orientation of sessions delivered.

All therapists (FT and TAU) were able to exercise their clinical judgement and make referrals to colleagues where they deemed this was clinically necessary, for example, access to local child and adolescent psychiatrists if medication or hospitalisation needed to be considered, or to colleagues with different assessment and therapeutic skills if specialised assessments or alternative treatments for problems other than self-harm were required. Without this condition local services would not have consented to randomisation of high risk participants.

Family Therapy: The funder specified a family intervention. There are many schools of family therapy (FT), but much of the outcome literature focuses on more structural/ behavioural model[1]. In the UK, the predominant model of family therapy is less behavioural and integrates different approaches and models allowing greater clinical flexibility than is usually the case with more narrowly defined models. The authors had available an existing FT manual derived from detailed observations of actual practice, the development and validation of which was funded by the Medical Research Council (MRC) to support trials of FT[2]. As this was a pragmatic trial the authors wished to evaluate a form of FT that was widely practised in the UK, and so adapted this existing manual to have an additional, significant focus on self-harm and risk assessment. This step also had the advantage of making the trial more acceptable to local clinical services.

The theoretical approach of the manual (available online[3]) allows for flexibility and integration of a broad range of conceptualizations from within the field of family therapy and other therapeutic approaches. It emphasizes the relational context of problems that families bring to therapy and that language, meaning, behaviour and emotions are all part of the change process. The manual permitted seeing the adolescent alone or in parallel sessions with a team member seeing the parents, and it encouraged the use of a reflecting team.

FT sessions lasted approximately 1¼ hours and were delivered over 6 months at approximately monthly intervals, though more frequently initially. The intention was to offer approximately 8 sessions according to clinical need. It was expected that some participants would receive fewer sessions due to drop-out or mutually agreed-upon termination of treatment. Equally, it was expected that some might receive more sessions (within the predefined 6-month period or extending beyond 6 months) where this was deemed clinically appropriate. Clinical data indicated that the average number of sessions offered in UK CAMHS clinics was around 6. Clinicians also told us that young people who had self-harmed were difficult to engage in longer-term treatment. As this was a pragmatic trial we designed an intervention that would be broadly equivalent in 'dose' (number of sessions) to TAU, and likely to be attended by participants and funded by the UK health care system. Delivering a more intensive intervention in real world settings would not have been feasible.

Wherever possible, and where consent was provided, sessions were video-recorded as this is part of good family therapy practice and facilitates supervision. In addition, this procedure facilitated central review of a selection of sessions to monitor adherence to the manual.

Qualified Family Therapists (those eligible for registration with the UK Council for Psychotherapy, UKCP, the highest level of accreditation in the UK) were appointed specifically to work on the trial. Therapists recruited to the study were expected to be at a senior grade usually requiring a minimum of 3 years post-qualifying practice. They were therefore senior and experienced practitioners.

Family therapists worked in teams of 3, one interviewing and two observing the family, and provided trial FT as a team for a cluster of CAMH services. Before the start of the trial SHIFT family therapists attended two days of group training and conducted a supervised pilot case before being assessed as ready to treat trial participants. One FT interviewing and two FTs observing the family, were expected to be present for most sessions. Once the trial had started the two senior trial family therapists (PB & IE) conducted monthly two-hour group supervision with each team. If a family therapist left during the trial, replacement therapists received 1-to-1 training with a senior SHIFT supervisor (PB or IE), a period of observation of team members' therapy, and a 1-to-1 session with the supervisor.

Therapist adherence to FT was ensured through training, use of the manual, regular peer supervision within each FT team, including videotape review, and regular external supervision. FT was monitored to ensure that

the number and timing of sessions was as planned. With consent, sessions were video-recorded to facilitate supervision. A random sample of videotapes (at least two per therapist) were independently rated to measure adherence to the core elements of the manualised family therapy, using a structured rating scale[4] (scored 0-5 for adherence, 0-6 for competence; higher scores indicate greater adherence / competence).

Strength of therapeutic alliance was reported by the young person, caregiver and SHIFT family therapist at the participant's third treatment session using the SOFTA questionnaire in which higher scores (ranging 0 to 80) represent greater alliance. The young person and therapist reported similar overall levels of alliance whilst caregivers consistently reported the highest levels of alliance, with overall alliance 57.9 (95% CI 56.7, 59.2, n=274) for the young person, 57.5 (95% CI 56.5, 58.4, n=293) for the family therapist and 65.4 (95% CI 64.4, 66.4, n=279) for the caregiver.

Contamination: The possibility of cross-arm contamination was considered during the design stage of the trial. Due to the nature of appointment scheduling, and the fact that this was family-specific therapy (that is, not a group intervention), there was little opportunity for participants to meet and discuss treatment, so contamination resulting from participant discussion was deemed very unlikely. In addition, SHIFT family therapists were prohibited from treating participants within the TAU arm for the duration of the trial. Any family-orientated clinical interventions in the TAU group were likely to be different from the trial FT intervention, which required adherence to the manual by fully-trained family therapists eligible for United Kingdom Council for Psychotherapy (UKCP) registration. SHIFT family therapists were also precluded from providing supervision to any TAU cases being seen within their own service. During the course of the study the SHIFT family therapy manual was embargoed.

Treatment as usual: TAU was the care offered by local CAMHS teams to adolescents referred following self-harm. This treatment was expected to be diverse and involve individual and/or family-orientated work, delivered by a range of practitioners with various theoretical orientations. As SHIFT is a pragmatic trial involving a number of collaborating CAMHS teams, the specification of TAU was not deemed possible or appropriate, although it was expected that CAMHS practitioners would be working in line with best practice as set out in several National Institute for Health and Care Excellence (NICE) guidelines (for example, guidance on self-harm and depression in childhood. TAU thus involved a wide range of treatment techniques and modalities (such as supportive counselling or cognitive behaviour therapy) that were not delivered to the FT group as part of the clinical intervention.

Table 2: Therapeutic orientation of all sessions and per participant

	Therapeutic orientation per session		Therapeutic orientation per participant (non-mutually exclusive)	
	**FT N=3207**	**TAU N=3466**	**FT N=415**	**TAU N=417**
**Therapy**				
SHIFT family therapy	2532 (79.0%)	0 (0.0%)	394 (94.9%)	0 (0.0%)
Supportive therapy/counselling	142 (4.4%)	871 (25.1%)	21 (5.1%)	158 (37.9%)
Cognitive behavioural therapy	79 (2.5%)	602 (17.4%)	10 (2.4%)	88 (21.1%)
Family work	35 (1.1%)	397 (11.5%)	9 (2.2%)	116 (27.8%)
Generic systemic family therapy	14 (0.4%)	371 (10.7%)	5 (1.2%)	87 (20.9%)
Communication skills/problem solving	0 (0.0%)	62 (1.8%)	0 (0.0%)	29 (7.0%)
Psycho-educational	22 (0.7%)	29 (0.8%)	6 (1.4%)	18 (4.3%)
Interpersonal therapy	5 (0.2%)	38 (1.1%)	1 (0.2%)	10 (2.4%)
Dialectical behaviour therapy	0 (0.0%)	30 (0.9%)	0 (0.0%)	8 (1.9%)
Psychodynamic	1 (0.0%)	131 (3.8%)	1 (0.2%)	5 (1.2%)
Other therapy	78 (2.4%)	168 (4.8%)	17 (4.1%)	28 (6.7%)
**Assessment**				
Mental state / Risk assessment	110 (3.4%)	137 (4.0%)	47 (11.3%)	58 (13.9%)
Other assessment / review	153 (4.8%)	434 (12.5%)	89 (21.4%)	161 (38.6%)
Medication review	26 (0.8%)	80 (2.3%)	13 (3.1%)	28 (6.7%)
Non therapy	2 (0.1%)	27 (0.8%)	1 (0.2%)	23 (5.5%)
**Unknown: per session / all sessions**	8 (0.2%)	89 (2.6%)	5 (1.2%)	27 (6.5%)
**No sessions attended**	--	--	16 (3.9%)	33 (7.9%)
**Missing all treatment data**	--	--	0 (0.0%)	45 (10.8%)

3. Full young person and caregiver scores at baseline

Table 3: Full baseline young person question scores and subscales

	FT N=415		TAU N=417		Total N=832	
**Outcome (range)**	**n**	**Mean (SD)**	**n**	**Mean (SD)**	**n**	**Mean (SD)**
**Total CDRS Score[1] (17 – 113)**	415	48.0 (14.19)	416	49.4 (13.29)	831	48.7 (13.76)
Not Depressed (<30)		45 (10.8%)		24 (5.8%)		69 (8.3%)
Mild Depression (30 - 42)		108 (26.0%)		108 (26.0%)		216 (26.0%)
Moderate Depression (43 - 57)		154 (37.1%)		168 (40.4%)		322 (38.7%)
Severe Depression (58 - 72)		91 (21.9%)		102 (24.5%)		193 (23.2%)
Very Severe Depression (>72)		17 (4.1%)		14 (3.4%)		31 (3.7%)
**BSS score[2] (0-38)**	408	10.8 (8.94)	408	10.4 (9.42)	816	10.6 (9.18)
**Suicide ideation (BSS Screening)** *Yes*		276 (67.6%)		267 (65.4%)		543 (66.5%)
**Total Hopelessness Score[3] (0 – 17)**	409	7.7 (4.30)	406	7.3 (4.19)	815	7.5 (4.25)
**SDQ[4]**						
**Total Difficulties Score (0 – 40)***	413	19.6 (5.70)	415	20.1 (5.60)	828	19.8 (5.65)
Close to average		84 (20.3%)		70 (16.9%)		154 (18.6%)
Slightly raised		58 (14.0%)		68 (16.4%)		126 (15.2%)
High		60 (14.5%)		44 (10.6%)		104 (12.6%)
Very high		211 (51.1%)		233 (56.1%)		444 (53.6%)
**Prosocial Score (0 – 10)**	414	7.1 (1.84)	415	7.2 (1.87)	829	7.2 (1.85)
**Emotional Problems Score (0 – 10)**	414	6.2 (2.34)	415	6.5 (2.31)	829	6.4 (2.33)
**Conduct Problems Score (0 – 10)**	414	3.9 (2.11)	415	4.0 (1.94)	829	3.9 (2.02)
**Hyperactivity Score (0 – 10)**	413	6.3 (2.22)	415	6.2 (2.17)	828	6.2 (2.20)
**Peer Problems Score (0 – 10)**	413	3.1 (1.87)	415	3.5 (2.08)	828	3.3 (1.98)
**Impact Score (0 – 10)**	413	3.3 (2.41)	414	3.5 (2.52)	827	3.4 (2.47)
**Externalising Score (0 – 20)**	413	10.2 (3.80)	415	10.1 (3.48)	828	10.2 (3.65)
**Internalising Score (0 – 20)**	413	9.4 (3.41)	415	10.0 (3.63)	828	9.7 (3.53)
**Total PQ-LES Score[5] (14-70)**	411	41.2 (9.37)	408	41.2 (9.45)	819	41.2 (9.41)
**Overall Self-Assessment Score (1-5)**	405	2.7 (1.04)	405	2.7 (1.00)	810	2.7 (1.02)
**Overall how has your life been**						
Very Poor		57 (14.1%)		53 (13.1%)		110 (13.6%)
Poor		107 (26.4%)		102 (25.2%)		209 (25.8%)
Fair		162 (40.0%)		167 (41.2%)		329 (40.6%)
Good		59 (14.6%)		70 (17.3%)		129 (15.9%)
Very Good		20 (4.9%)		13 (3.2%)		33 (4.1%)
**ICU[6]**	410		403		813	
**ICU Total Score (0 - 72)**		28.2 (9.10)		28.5 (9.09)		28.4 (9.09)
**ICU Callousness (0 - 33)**		8.3 (4.61)		8.6 (4.99)		8.5 (4.80)
**ICU Uncaring Score (0 - 24)**		11.0 (4.65)		10.8 (4.57)		10.9 (4.61)
**ICU Unemotional Score (0 - 15)**		8.9 (2.82)		9.1 (3.09)		9.0 (2.96)

Outcome (range)	FT N=415 n	FT N=415 Mean (SD)	TAU N=417 n	TAU N=417 Mean (SD)	Total N=832 n	Total N=832 Mean (SD)
**McMaster FAD[7] (1 – 4)**						
**Overall FAD Score**	404	2.5 (0.33)	405	2.4 (0.36)	809	2.5 (0.35)
**General Functioning**	410	2.5 (0.53)	408	2.5 (0.56)	818	2.5 (0.54)
Unhealthy (≥2.0)		354 (86.3%)		339 (83.1%)		693 (84.7%)
**Behaviour Control Subscale**	413	2.1 (0.38)	409	2.1 (0.37)	822	2.1 (0.38)
Unhealthy (≥1.9)		319 (77.2%)		311 (76.0%)		630 (76.6%)
**Affective Involvement Subscale**	412	2.5 (0.48)	409	2.5 (0.49)	821	2.5 (0.48)
Unhealthy (≥2.1)		345 (83.7%)		341 (83.4%)		686 (83.6%)
**Affective Responsiveness Subscale**	410	2.6 (0.48)	408	2.6 (0.51)	818	2.6 (0.50)
Unhealthy (≥2.2)		343 (83.7%)		335 (82.1%)		678 (82.9%)
**Roles Subscale**	412	2.5 (0.35)	409	2.5 (0.37)	821	2.5 (0.36)
Unhealthy (≥2.3)		310 (75.2%)		309 (75.6%)		619 (75.4%)
**Communication Subscale**	413	2.6 (0.37)	409	2.6 (0.38)	822	2.6 (0.37)
Unhealthy (≥2.2)		360 (87.2%)		343 (83.9%)		703 (85.5%)
**Problem Solving Subscale**	410	2.5 (0.47)	409	2.5 (0.53)	819	2.5 (0.50)
Unhealthy (≥2.2)		331 (80.7%)		306 (74.8%)		637 (77.8%)

[1] CDRS - Children's Depression Rating Scale: Higher scores represent greater levels of depression.
[2] BSS - Beck Scale for Suicide Ideation: Higher scores indicate a higher level of suicide ideation. Median presented as scores considerably skewed and zero inflated at follow up.
[3] Hopelessness Scale for Children: Higher scores reflect greater hopelessness or negative expectations toward the future.
[4] SDQ - Strengths and Difficulties Questionnaire: Higher scores represent greater levels of concern in all categories bar prosocial where the reverse is true.
[5] PQ-LES - Paediatric quality of Life Enjoyment and Satisfaction measure: Higher scores indicative of greater enjoyment and satisfaction.
[6] ICU - Inventory of Callous Unemotional Traits: Higher scores represent higher callous and unemotional traits.
[7] FAD - McMaster Family Assessment Device: Higher scores are indicative of poorer family functioning.

Table 4: Full baseline caregiver question scores and subscales

	FT N=415		TAU N=417		Total N=832	
**Outcome (range)**	**n**	**Mean (SD)**	**n**	**Mean (SD)**	**n**	**Mean (SD)**
**SDQ[1]**						
**Total Difficulties Score (0 – 40)**	412	19.4 (6.56)	415	19.8 (6.83)	827	19.6 (6.69)
Close to average		78 (18.9%)		86 (20.7%)		164 (19.8%)
Slightly raised		51 (12.4%)		36 (8.7%)		87 (10.5%)
High		67 (16.3%)		66 (15.9%)		133 (16.1%)
Very high		216 (52.4%)		227 (54.7%)		443 (53.6%)
**Prosocial Score (0 – 10)**	414	6.3 (2.33)	416	6.3 (2.30)	830	6.3 (2.31)
**Emotional Problems Score (0 – 10)**	414	6.2 (2.39)	415	6.2 (2.60)	829	6.2 (2.49)
**Conduct Problems Score (0 – 10)**	413	4.2 (2.42)	416	4.3 (2.47)	829	4.2 (2.44)
**Hyperactivity Score (0 – 10)**	414	5.5 (2.48)	415	5.7 (2.56)	829	5.6 (2.52)
**Peer Problems Score (0 – 10)**	413	3.5 (2.09)	415	3.6 (2.14)	828	3.6 (2.11)
**Impact Score (0 – 10)**	410	4.4 (2.73)	413	4.3 (2.73)	823	4.4 (2.73)
**Externalising Score (0 – 20)**	413	9.7 (4.30)	415	10.0 (4.50)	828	9.9 (4.40)
**Internalising Score (0 – 20)**	413	9.8 (3.69)	415	9.8 (4.02)	828	9.8 (3.86)
**GHQ-12 Score. Likert scale[2] (0 – 36)**	414	17.7 (7.06)	415	18.6 (7.24)	829	18.2 (7.16)
**ICU[3]**	413		415		828	
**ICU Total Score (0 - 72)**		32.6 (11.59)		32.9 (11.43)		32.8 (11.50)
**ICU Callousness (0 - 33)**		10.4 (6.62)		10.7 (6.68)		10.5 (6.65)
**ICU Uncaring Score (0 - 24)**		14.5 (4.85)		14.5 (4.82)		14.5 (4.83)
**ICU Unemotional Score (0 - 15)**		7.6 (3.08)		7.7 (3.08)		7.7 (3.07)
**McMaster FAD[4] (1 – 4)**						
**Overall FAD Score**	408	2.2 (0.36)	415	2.2 (0.36)	823	2.2 (0.36)
**General Functioning**	415	2.3 (0.48)	416	2.3 (0.47)	831	2.3 (0.47)
Unhealthy (≥2.0)		314 (75.7%)		316 (76.0%)		630 (75.8%)
**Behaviour Control Subscale**	411	1.8 (0.41)	416	1.8 (0.41)	827	1.8 (0.41)
Unhealthy (≥1.9)		197 (47.9%)		226 (54.3%)		423 (51.1%)
**Affective Involvement Subscale**	412	2.2 (0.45)	415	2.3 (0.51)	827	2.2 (0.48)
Unhealthy (≥2.1)		270 (65.5%)		272 (65.5%)		542 (65.5%)
**Affective Responsiveness Subscale**	411	2.1 (0.55)	415	2.1 (0.57)	826	2.1 (0.56)
Unhealthy (≥2.2)		195 (47.4%)		197 (47.5%)		392 (47.5%)
**Roles Subscale**	415	2.5 (0.42)	415	2.5 (0.42)	830	2.5 (0.42)
Unhealthy (≥2.3)		304 (73.3%)		309 (74.5%)		613 (73.9%)
**Communication Subscale**	413	2.3 (0.44)	415	2.3 (0.41)	828	2.3 (0.43)
Unhealthy (≥2.2)		248 (60.0%)		255 (61.4%)		503 (60.7%)
**Problem Solving Subscale**	411	2.2 (0.48)	416	2.2 (0.48)	827	2.2 (0.48)
Unhealthy (≥2.2)		234 (56.9%)		249 (59.9%)		483 (58.4%)
**Family Questionnaire[5]**	415		416		831	
**Total Score (20 – 80)**		52.9 (10.67)		52.9 (10.85)		52.9 (10.75)
**Emotional Over Involvement (10 – 40)**		27.2 (4.93)		27.2 (5.03)		27.2 (4.98)
**Criticism (10 – 40)**		25.7 (6.97)		25.7 (7.06)		25.7 (7.01)

[1] SDQ - Strengths and Difficulties Questionnaire: Higher scores represent greater levels of concern in all categories bar prosocial where the reverse is true.
[2] GHQ-12 - General Health Questionnaire-12: Higher scores are indicative of greater psychological distress.
[3] ICU - Inventory of Callous Unemotional Traits: Higher scores represent higher callous and unemotional traits.
[4] FAD - McMaster Family Assessment Device: Higher scores are indicative of poorer family functioning.
[5] Family questionnaire: Higher scores indicate greater levels of expressed emotion.

4. Full young person and caregiver scores on secondary outcomes at 12 and 18 months

Table 5: Mean young-person questionnaire scores with 95% Confidence Intervals for young person questionnaire outcomes at 12 and 18 months, adjusted for baseline score and covariates with multiple imputation[i]

	Young person[h]					
	12 months			18 Months		
**Outcome**	**FT, mean (95% CI), SE**	**TAU, mean (95% CI), SE**	**Difference[g], mean (95% CI), SE, p-value**	**FT, mean (95% CI), SE**	**TAU, mean (95% CI), SE**	**Difference[g], mean (95% CI), SE, p-value**
**CDRS[a]**	33.2 (30.4, 36.1), SE=1.46	33.9 (30.8, 37.0), SE=1.57	-0.6 (-3.1, 1.9), SE=1.27, p=0.62	30.6 (27.6, 33.6), SE=1.50	31.6 (28.7, 34.5), SE=1.46	-1.0 (-3.5, 1.5), SE=1.26, p=0.43
**PQ-LES[b]**	49.9 (47.7, 52.1), SE=1.12	48.8 (46.5, 51.0), SE=1.13	1.1 (-0.5, 2.7), SE=0.82, p=0.18	50.6 (48.4, 52.8), SE=1.12	50.4 (48.1, 52.8), SE=1.20	0.1 (-1.9, 2.1), SE=1.02, p=0.90
**Hopelessness[c]**	4.8 (4.0, 5.6), SE=0.40	5.1 (4.3, 6.0), SE=0.43	-0.3 (-1.1, 0.4), SE=0.37, p=0.38	4.4 (3.6, 5.2), SE=0.42	4.6 (3.7, 5.4), SE=0.43	-0.2 (-0.9, 0.5), SE=0.36, p=0.63
**SDQ[d]**						
Total Difficulties	14.8 (13.4, 16.1), SE=0.69	15.5 (14.1, 16.9), SE=0.70	-0.7 (-1.8, 0.4), SE=0.54, p=0.19	13.3 (12.0, 14.6), SE=0.67	14.1 (12.7, 15.5), SE=0.71	-0.8 (-2.0, 0.4), SE=0.61, p=0.18
Prosocial Score	7.7 (7.3, 8.1), SE=0.19	7.3 (6.9, 7.7), SE=0.19	**0.4 (0.1, 0.7), SE=0.15, p=0.0064**	7.8 (7.4, 8.1), SE=0.18	7.4 (7.1, 7.8), SE=0.19	**0.3 (0.0, 0.7), SE=0.16, p=0.034**
Emotional Problems Score	4.4 (3.8, 4.9), SE=0.28	4.3 (3.7, 4.8), SE=0.28	0.1 (-0.4, 0.5), SE=0.22, p=0.81	3.7 (3.2, 4.3), SE=0.27	4.0 (3.5, 4.6), SE=0.28	-0.3 (-0.8, 0.2), SE=0.24, p=0.20
Conduct Problems Score	2.7 (2.4, 3.1), SE=0.19	2.9 (2.5, 3.3), SE=0.19	-0.2 (-0.5, 0.1), SE=0.15, p=0.21	2.4 (2.0, 2.7), SE=0.18	2.4 (2.0, 2.8), SE=0.19	0.0 (-0.3, 0.3), SE=0.16, p=0.90
Hyperactivity Score	5.0 (4.5, 5.5), SE=0.25	5.3 (4.8, 5.9), SE=0.26	-0.3 (-0.7, 0.1), SE=0.20, p=0.098	4.5 (4.1, 5.0), SE=0.24	4.9 (4.4, 5.4), SE=0.26	-0.4 (-0.8, 0.1), SE=0.22, p=0.11
Peer Problems Score	2.6 (2.2, 3.0), SE=0.20	2.9 (2.5, 3.3), SE=0.20	-0.3 (-0.6, 0.0), SE=0.16, p=0.050	2.5 (2.1, 2.9), SE=0.20	2.8 (2.4, 3.2), SE=0.21	-0.3 (-0.6, 0.1), SE=0.18, p=0.13
Impact Score	2.0 (1.5, 2.6), SE=0.27	2.7 (2.2, 3.2), SE=0.25	**-0.7 (-1.1, -0.2), SE=0.22, p=0.0033**	1.9 (1.3, 2.5), SE=0.29	2.2 (1.6, 2.8), SE=0.30	-0.3 (-0.8, 0.2), SE=0.26, p=0.22
Externalising Score	7.7 (7.0, 8.5), SE=0.38	8.2 (7.5, 9.0), SE=0.39	-0.5 (-1.1, 0.1), SE=0.30, p=0.082	6.9 (6.2, 7.6), SE=0.37	7.2 (6.5, 8.0), SE=0.39	-0.3 (-1.0, 0.3), SE=0.33, p=0.32
Internalising Score	7.0 (6.2, 7.8), SE=0.40	7.2 (6.4, 8.0), SE=0.41	-0.2 (-0.9, 0.4), SE=0.32, p=0.48	6.3 (5.5, 7.1), SE=0.39	6.8 (6.0, 7.6), SE=0.41	-0.5 (-1.2, 0.2), SE=0.36, p=0.14
**McMaster FAD[e]**						
Overall FAD Score	2.3 (2.2, 2.3), SE=0.04	2.3 (2.2, 2.4), SE=0.04	-0.0 (-0.1, 0.0), SE=0.03, p=0.67	2.2 (2.1, 2.3), SE=0.04	2.2 (2.1, 2.3), SE=0.04	-0.0 (-0.1, 0.1), SE=0.03, p=0.93
General Functioning	2.2 (2.1, 2.3), SE=0.06	2.2 (2.1, 2.4), SE=0.06	-0.0 (-0.1, 0.1), SE=0.05, p=0.74	2.2 (2.1, 2.3), SE=0.06	2.2 (2.0, 2.3), SE=0.06	0.0 (-0.1, 0.1), SE=0.05, p=0.81
Behaviour Control	2.0 (1.9, 2.0), SE=0.04	1.9 (1.9, 2.0), SE=0.04	0.0 (-0.0, 0.1), SE=0.03, p=0.63	1.9 (1.8, 2.0), SE=0.04	1.9 (1.8, 2.0), SE=0.05	-0.0 (-0.1, 0.1), SE=0.04, p=0.57

	Young person[h]					
	12 months			18 Months		
**Outcome**	**FT, mean (95% CI), SE**	**TAU, mean (95% CI), SE**	**Difference[g], mean (95% CI), SE, p-value**	**FT, mean (95% CI), SE**	**TAU, mean (95% CI), SE**	**Difference[g], mean (95% CI), SE, p-value**
Affective Involvement	2.4 (2.3, 2.5), SE=0.05	2.4 (2.3, 2.5), SE=0.05	-0.1 (-0.1, 0.0), SE=0.04, p=0.22	2.3 (2.2, 2.4), SE=0.05	2.4 (2.3, 2.5), SE=0.06	-0.1 (-0.2, 0.0), SE=0.05, p=0.16
Affective Responsiveness	2.4 (2.3, 2.5), SE=0.06	2.4 (2.3, 2.6), SE=0.06	-0.0 (-0.1, 0.1), SE=0.05, p=0.66	2.4 (2.3, 2.5), SE=0.06	2.4 (2.3, 2.5), SE=0.06	-0.0 (-0.1, 0.1), SE=0.05, p=0.94
Roles	2.3 (2.2, 2.4), SE=0.04	2.3 (2.2, 2.4), SE=0.04	-0.0 (-0.1, 0.0), SE=0.03, p=0.46	2.2 (2.2, 2.3), SE=0.04	2.2 (2.2, 2.3), SE=0.04	-0.0 (-0.1, 0.1), SE=0.04, p=0.87
Communication	2.4 (2.3, 2.5), SE=0.04	2.4 (2.3, 2.4), SE=0.04	0.0 (-0.0, 0.1), SE=0.04, p=0.26	2.3 (2.2, 2.4), SE=0.05	2.3 (2.2, 2.4), SE=0.05	0.0 (-0.1, 0.1), SE=0.04, p=0.78
Problem Solving	2.3 (2.2, 2.4), SE=0.05	2.3 (2.2, 2.4), SE=0.05	-0.0 (-0.1, 0.1), SE=0.04, p=0.81	2.2 (2.1, 2.3), SE=0.05	2.2 (2.1, 2.3), SE=0.05	-0.0 (-0.1, 0.1), SE=0.05, p=0.61
**BSS**[f] Suicide ideation screening: *yes*	0.26 (0.17, 0.36), SE=0.05	0.36 (0.25, 0.46), SE=0.05	0.64 (0.44, 0.94)**, p=0.024**	0.22 (0.14, 0.31), SE=0.04	0.28 (0.18, 0.37), SE=0.05	0.76 (0.49, 1.16), p=0.20

[a] CDRS - Children's Depression Rating Scale: Higher scores represent greater levels of depression.
[b] PQ-LES - Paediatric quality of Life Enjoyment and Satisfaction measure: Higher scores indicative of greater enjoyment and satisfaction.
[c] Hopelessness scale for Children: Higher scores reflect greater hopelessness or negative expectations toward the future.
[d] SDQ - Strengths and Difficulties Questionnaire: Higher scores represent greater levels of concern in all categories bar prosocial where the reverse is true.
[e] FAD - McMaster Family Assessment Device: Higher scores are indicative of poorer family functioning.
[f] BSS - Beck Scale for Suicide Ideation screening – binary outcome. FT and TAU estimates reflect the proportion with suicide ideation, the difference represents the odds ratio.
[g] Difference: FT – TAU for continuous outcomes, Odds ratio for binary BSS outcome.
[h] Estimates derived using multiple imputation of missing data. Complete data was available for a maximum of 261/415 FT and 204/417 TAU participants at 12 months, and 213/415 FT and 182/417 TAU participants at 18 months for participants who had completed the young person booklet. Note slightly less complete data was available for the CDRS, collected through researcher interview.
[I] Multiple imputation, assuming data were "Missing at Random", was used to account for missing questionnaire data[5]; the complete case formed a sensitivity analysis. Using the Markov Chain Monte Carlo method, results from 33 imputations (according to 33% missing data across all time-points) were combined using Rubin's rules[6]. Missing values were imputed separately for each questionnaire, incorporating available scores at baseline, 12 and 18-months, covariates and treatment.

Table 6: Mean young-person questionnaire scores with 95% Confidence Intervals for caregiver questionnaire outcomes at 12 and 18 months, adjusted for baseline score and covariates with multiple imputation[g]

	Caregiver[f]					
	12 months			18 Months		
**Outcome**	**FT, mean (95% CI), SE**	**TAU, mean (95% CI), SE**	**Difference[e], mean (95% CI), SE, p-value**	**FT, mean (95% CI), SE**	**TAU, mean (95% CI), SE**	**Difference[e], mean (95% CI), SE, p-value**
**GHQ-12[a]**	12.8 (11.6, 14.0), SE=0.61	13.5 (12.3, 14.8), SE=0.65	-0.7 (-1.8, 0.3), SE=0.54, p=0.19	13.0 (11.8, 14.2), SE=0.62	13.2 (11.8, 14.6), SE=0.71	-0.2 (-1.3, 0.9), SE=0.57, p=0.73
**Family Questionnaire (3 & 6m)[b]**						
Total Score	50.9 (49.0, 52.7), SE=0.96	50.2 (48.3, 52.0), SE=0.95	0.7 (-0.7, 2.1), SE=0.73, p=0.34	47.4 (45.3, 49.4), SE=1.06	48.8 (46.7, 50.9), SE=1.07	-1.4 (-3.3, 0.5), SE=0.94, p=0.14
Emotional Sub score	25.9 (24.9, 26.9), SE=0.50	25.5 (24.6, 26.5), SE=0.50	0.4 (-0.4, 1.1), SE=0.38, p=0.35	23.8 (22.7, 24.9), SE=0.55	24.4 (23.3, 25.5), SE=0.56	-0.6 (-1.6, 0.4), SE=0.50, p=0.26
Criticism Sub score	25.0 (23.9, 26.1), SE=0.55	24.7 (23.6, 25.8), SE=0.55	0.3 (-0.5, 1.2), SE=0.43, p=0.44	23.6 (22.4, 24.8), SE=0.61	24.4 (23.2, 25.6), SE=0.61	-0.9 (-1.9, 0.2), SE=0.54, p=0.12
**SDQ[c]**						
Total Difficulties	14.1 (12.7, 15.5), SE=0.72	15.4 (14.0, 16.8), SE=0.71	**-1.3 (-2.4, -0.2), SE=0.56, p=0.026**	13.2 (11.9, 14.6), SE=0.68	14.9 (13.3, 16.4), SE=0.79	**-1.6 (-2.9, -0.4), SE=0.65, p=0.013**
Prosocial Score	6.9 (6.5, 7.3), SE=0.21	6.8 (6.4, 7.2), SE=0.22	0.1 (-0.3, 0.4), SE=0.17, p=0.60	7.1 (6.6, 7.5), SE=0.22	6.9 (6.5, 7.4), SE=0.22	0.2 (-0.2, 0.5), SE=0.18, p=0.39
Emotional Problems Score	4.0 (3.4, 4.5), SE=0.28	4.5 (3.9, 5.1), SE=0.29	**-0.5 (-1.0, -0.1), SE=0.23, p=0.017**	3.6 (3.1, 4.2), SE=0.28	4.2 (3.6, 4.8), SE=0.30	**-0.6 (-1.1, -0.1), SE=0.25, p=0.022**
Conduct Problems Score	3.1 (2.7, 3.4), SE=0.20	3.3 (2.9, 3.7), SE=0.20	-0.3 (-0.6, 0.0), SE=0.17, p=0.093	2.8 (2.4, 3.2), SE=0.20	3.1 (2.7, 3.5), SE=0.21	**-0.3 (-0.6, -0.0), SE=0.16, p=0.050**
Hyperactivity Score	4.3 (3.8, 4.7), SE=0.24	4.4 (3.9, 4.9), SE=0.25	-0.1 (-0.5, 0.3), SE=0.19, p=0.55	4.3 (3.8, 4.8), SE=0.24	4.5 (4.0, 5.1), SE=0.28	-0.2 (-0.6, 0.2), SE=0.22, p=0.35
Peer Problems Score	2.9 (2.5, 3.3), SE=0.21	3.2 (2.8, 3.6), SE=0.21	**-0.3 (-0.7, -0.0), SE=0.17, p=0.037**	2.6 (2.2, 3.0), SE=0.20	3.1 (2.7, 3.6), SE=0.23	**-0.5 (-0.9, -0.1), SE=0.20, p=0.0092**
Impact Score	2.3 (1.7, 2.9), SE=0.31	2.9 (2.2, 3.7), SE=0.37	**-0.7 (-1.3, -0.1), SE=0.30, p=0.031**	2.4 (1.7, 3.0), SE=0.32	2.6 (1.9, 3.3), SE=0.36	-0.3 (-0.9, 0.3), SE=0.31, p=0.38
Externalising Score	7.3 (6.6, 8.0), SE=0.36	7.7 (6.9, 8.4), SE=0.38	-0.4 (-1.0, 0.2), SE=0.31, p=0.18	7.0 (6.2, 7.8), SE=0.39	7.6 (6.9, 8.4), SE=0.39	**-0.7 (-1.3, -0.0), SE=0.32, p=0.045**
Internalising Score	6.8 (6.0, 7.7), SE=0.43	7.7 (6.9, 8.6), SE=0.43	**-0.9 (-1.5, -0.2), SE=0.34, p=0.011**	6.2 (5.4, 7.0), SE=0.41	7.3 (6.3, 8.2), SE=0.48	**-1.1 (-1.9, -0.3), SE=0.39, p=0.0074**
**McMaster FAD[d]**						
Overall FAD Score	2.1 (2.0, 2.1), SE=0.03	2.1 (2.0, 2.2), SE=0.03	-0.0 (-0.1, 0.0), SE=0.03, p=0.080	2.0 (2.0, 2.1), SE=0.03	2.1 (2.0, 2.1), SE=0.03	-0.0 (-0.1, 0.0), SE=0.03, p=0.64
General Functioning	2.1 (2.0, 2.2), SE=0.04	2.1 (2.0, 2.2), SE=0.04	-0.0 (-0.1, 0.0), SE=0.04, p=0.24	2.0 (1.9, 2.1), SE=0.05	2.1 (2.0, 2.2), SE=0.04	-0.0 (-0.1, 0.0), SE=0.04, p=0.21
Behaviour Control	1.7 (1.6, 1.8), SE=0.04	1.7 (1.6, 1.8), SE=0.04	-0.0 (-0.1, 0.1), SE=0.03, p=0.87	1.7 (1.6, 1.7), SE=0.04	1.7 (1.6, 1.8), SE=0.04	-0.0 (-0.1, 0.1), SE=0.03, p=0.82
Affective Involvement	2.1 (2.0, 2.2), SE=0.04	2.1 (2.1, 2.2), SE=0.04	-0.1 (-0.1, 0.0), SE=0.03, p=0.065	2.1 (2.0, 2.2), SE=0.04	2.1 (2.0, 2.2), SE=0.05	-0.0 (-0.1, 0.1), SE=0.05, p=0.57

	Caregiver[f]					
	12 months			18 Months		
**Outcome**	**FT, mean (95% CI), SE**	**TAU, mean (95% CI), SE**	**Difference[e], mean (95% CI), SE, p-value**	**FT, mean (95% CI), SE**	**TAU, mean (95% CI), SE**	**Difference[e], mean (95% CI), SE, p-value**
Affective Responsiveness	2.0 (1.9, 2.1), SE=0.04	2.0 (1.9, 2.1), SE=0.05	-0.0 (-0.1, 0.1), SE=0.04, p=0.67	2.0 (1.9, 2.1), SE=0.05	1.9 (1.9, 2.0), SE=0.05	0.0 (-0.1, 0.1), SE=0.04, p=0.43
Roles	2.4 (2.3, 2.4), SE=0.03	**2.5 (2.4, 2.5), SE=0.04**	**-0.1 (-0.2, -0.0), SE=0.03, p=0.0020**	2.4 (2.3, 2.4), SE=0.04	2.4 (2.3, 2.5), SE=0.04	-0.0 (-0.1, 0.1), SE=0.03, p=0.73
Communication	2.1 (2.0, 2.2), SE=0.04	2.2 (2.1, 2.3), SE=0.04	-0.0 (-0.1, 0.0), SE=0.03, p=0.13	2.1 (2.0, 2.2), SE=0.04	2.1 (2.1, 2.2), SE=0.04	-0.0 (-0.1, 0.0), SE=0.04, p=0.29
Problem Solving	2.0 (2.0, 2.1), SE=0.04	2.1 (2.0, 2.2), SE=0.04	-0.0 (-0.1, 0.0), SE=0.03, p=0.26	2.0 (1.9, 2.1), SE=0.04	2.0 (1.9, 2.1), SE=0.04	-0.0 (-0.1, 0.1), SE=0.04, p=0.70

[a] GHQ-12 - General Health Questionnaire-12: Higher scores are indicative of greater psychological distress.
[b] Family Questionnaire: Higher scores indicate greater levels of expressed emotion.
[c] SDQ - Strengths and Difficulties Questionnaire: Higher scores represent greater levels of concern in all categories bar prosocial where the reverse is true.
[d] FAD - McMaster Family Assessment Device: Higher scores are indicative of poorer family functioning.
[e] Difference: FT – TAU
[f] Estimates derived using multiple imputation of missing data. Complete data were available for a maximum of 254/415 FT and 195/417 TAU participants at 12 months, and 220/415 FT and 176/417 TAU participants at 18 months for participants who had completed caregiver booklet.
[g] Multiple imputation, assuming data were "Missing at Random", was used to account for missing questionnaire data[5]; the complete case formed a sensitivity analysis. Using the Markov Chain Monte Carlo method, results from 33 imputations (according to 33% missing data across all time-points) were combined using Rubin's rules[6]. Missing values were imputed separately for each questionnaire, incorporating available scores at baseline, 12 and 18-months, covariates and treatment.

5. Predictive and process measures

a. Moderator analysis:
Covariates and responses to all baseline questionnaires were explored for moderation. A 5% significance level was used to identify moderation through the interaction of the potential moderator with treatment, irrespective of the main effect of the potential moderator. Analysis was of the ITT population to availability of data (complete case) for each proposed moderator. Proposed moderators included:

- Covariates: age (11-14, 15-17), trust, referral from hospital (yes, no), baseline number of self-harm episodes (2, >2), type of index self-harm episode (self-poisoning, self-injury, combined), sex (male, female);
- Young person baseline questionnaire responses: BSS; CDRS R; PQ-LES-Q, Hopelessness;
- Caregiver baseline questionnaire responses: Family questionnaire – criticism, emotional over-involvement; GHQ-12;
- Young person and caregiver baseline questionnaire responses: McMaster FAD - affective involvement, affective responsiveness, behaviour control communication, general functioning, problem-solving, roles, total score; ICU - callousness, uncaring, unemotional, total score; SDQ - conduct problems, emotional problems, externalising, hyperactivity, impact, internalising, peer problems, prosocial, total difficulties;
- Categorised baseline questionnaire responses: young person BSS, to indicate whether suicidal ideation was present; young person CDRS-R, to indicate whether depression was present; young person and caregiver McMaster FAD, to indicate whether family functioning was healthy or unhealthy - affective involvement, affective responsiveness, behaviour control communication, general functioning, problem-solving, roles.

b. Mediator analysis:
Mediator analysis included both Complier Average Causal Effect (CACE) analysis to model the causal effect of FT receipt on the primary outcome, and further mediation analysis to identify process variables and other potential mediators that influence engagement with and benefit from treatment.

b.1 Complier average causal effect analysis: Methods and key results of the CACE analysis are presented in the main paper. Additional illustrations are presented in Supplementary Figure 1 presenting the time to self-harm in each arm by receipt of formal systemic family therapy, and by arm. TAU participants with missing treatment data had the highest overall self-harm rate with a primary outcome event reported in 15/45 (33.3%) participants. The lowest repetition rate was reported in 3/21 (14.3%) participants allocated to receive FT who attended no FT sessions although this also comprised the smallest group. The repetition rate in participants allocated to and who attended SHIFT Family Therapy was 29.2% (115/394), whilst for those in the TAU arm not attending Family Therapy, the repetition rate was lower at 24.6% (70/285). Furthermore, in participants allocated to TAU who attended Family Therapy as part of their usual care, the repetition rate was lower again at 20.7% (18/87). Considering attendance at Family Therapy sessions irrespective of randomisation, the repetition rate in those receiving at least one Family Therapy session was 27.7% (133/481), and 23.9% (73/306) in those who did not.

Figure 1: Kaplan-Meier plot of time to self-harm by Randomised Treatment group and receipt of Family Therapy

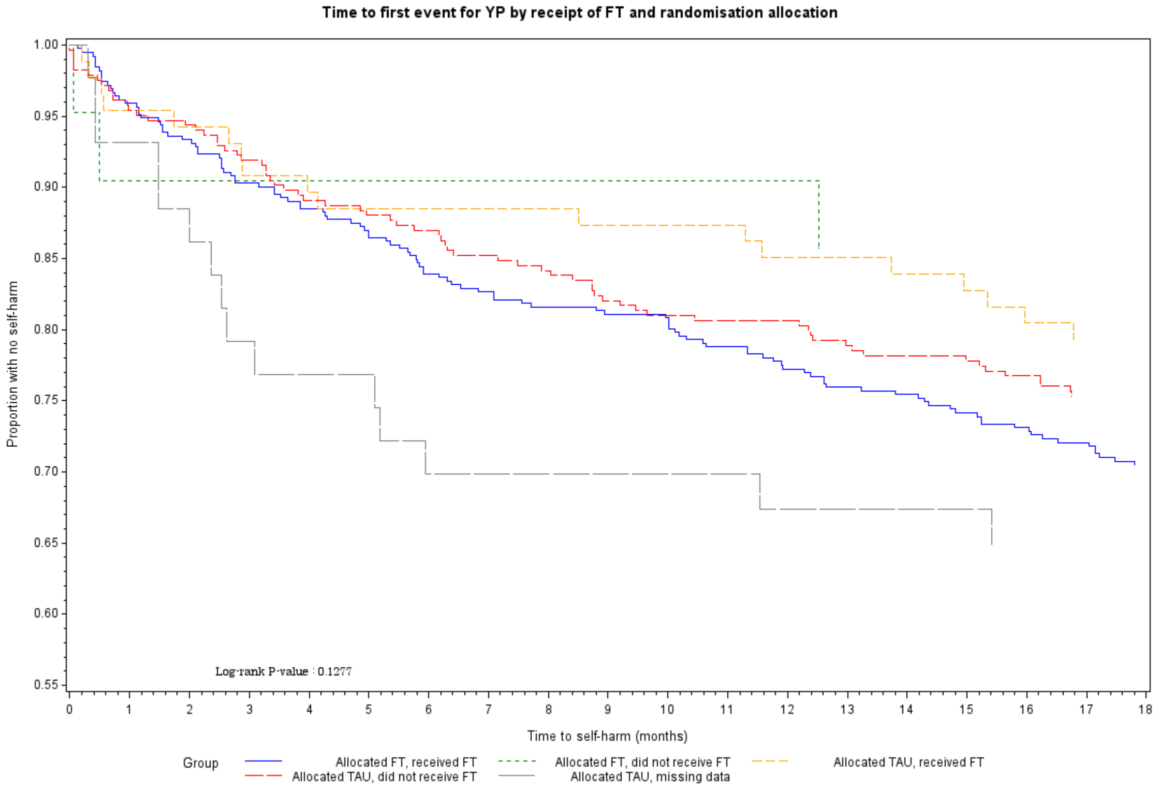

b.2 Further mediation

Methods: Process variables explored as potential mediators included the overall number of sessions attended, the use of psychotropic medications and therapist characteristics. Questionnaire responses were also investigated but results are not presented here due to the significant proportion of missing data.
The Baron and Kenny steps[7] were employed to explore mediation:

- Step 1 – establish an effect of randomisation (X) on outcome (Y) that may be mediated
- Step 2 – establish that there is an effect of randomisation (X) on the mediator (M)
- Step 3 – establish that there is an effect of mediator (M) on outcome (Y) while controlling for randomisation (X)

Following these steps, complete mediation is the case in which randomisation no longer affects the outcome (time to self-harm) after the mediator has been controlled. Partial mediation is the case in which the path from randomisation to time to self-harm is reduced in absolute size but is still different from zero when the mediator is introduced.
Steps 1 and 3 were fitted using a Cox proportional hazards model adjusted for covariates. For the number of sessions, Step 2 was fitted using a linear regression model containing randomised treatment and covariates; for psychotropic medications and therapist characteristics, logistic regression was used containing randomised treatment and covariates apart from NHS trust (owing to lack of convergence).

Results: Overall, young people on a psychotropic medication during follow-up were more likely to self-harm than those who were not prescribed such drugs: 41 out of 104 (39.4%) young people on a medication engaged in self-harm, compared with 164 out of 670 (24.5%) not on medication (Supplementary Table 6). In the FT arm, rates of self-harm were higher in young people whose lead therapist had been working in CAMHS for 4 or more years than in those whose lead therapist had been working for less than 4 years, with repetition observed for 77 (27.8%) and 9 (20.9%) participants, respectively. Conversely, in the TAU arm, rates of self-harm were lower among participants seen by more experienced therapists than among those seen by less experienced therapists, with repetition observed for 24 (19.7%) and 12 (26.7%) participants, respectively. In both arms, participants who self-harmed attended more sessions overall (median 7 sessions) than those who did not (median 5 sessions).

Table 7: Summary of self-harm event by potential process mediator and treatment arm

Potential Mediator	Primary outcome event reported – Yes (self-harm), No (no self-harm)					
	Family Therapy		Treatment as Usual		Total	
	Yes	No	Yes	No	Yes	No
**Young Person on any psychotropic medications**						
Yes (n=104)	18 (40.9%)	26 (59.1%)	23 (38.3%)	37 (61.7%)	41 (39.4%)	63 (60.6%)
No (n=670)	100 (27.2%)	267 (72.8%)	64 (21.1%)	239 (78.9%)	164 (24.5%)	506 (75.5%)
Missing (n=58)	0 (0.0%)	4 (100.0%)	16 (29.6%)	38 (70.4%)	16 (27.6%)	42 (72.4%)
Total (n=832)	118 (28.4%)	297 (71.6%)	103 (24.7%)	314 (75.3%)	221 (26.6%)	611 (73.4%)
**Years spent working in CAMHS**						
<4 years (n=88)	9 (20.9%)	34 (79.1%)	12 (26.7%)	33 (73.3%)	21 (23.9%)	67 (76.1%)
≥ 4 years (n=399)	77 (27.8%)	200 (72.2%)	24 (19.7%)	98 (80.3%)	101 (25.3%)	298 (74.7%)
Missing (n=345)	32 (33.7%)	63 (66.3%)	67 (26.8%)	183 (73.2%)	99 (28.7%)	246 (71.3%)
Total (n=832)	118 (28.4%)	297 (71.6%)	103 (24.7%)	314 (75.3%)	221 (26.6%)	611 (73.4%)
**Overall number of sessions attended by anyone**						
N	118	297	88	284	206	581
Mean (SD)	9.9 (10.32)	6.8 (5.32)	12.9 (15.18)	8.2 (13.25)	11.2 (12.68)	7.5 (10.03)
Median (Range)	7.0 (0, 70)	6.0 (0, 47)	8.0 (0, 90)	4.0 (0, 163)	7.0 (0, 90)	5.0 (0, 163)

Following the Baron and Kenny steps, there was no evidence that any of the variables investigated formally mediated the effect of treatment on the time to self-harm, largely because of lack of evidence of a treatment effect (step 1) (Supplementary Table 7).

However, there was good evidence (step 2) of an association between randomised treatment and psychotropic medication use during follow-up (less medication in FT: OR 0.6; $p = 0.016$) and strong evidence (step 3) that the use of psychotropic medication was associated with an increased risk of self-harm (HR 2.10; $p < 0.0001$). There was, however, no evidence of a direct treatment effect despite the positive association with FT and the mediator (step 3).

There was weak evidence (step 2) of an association between randomised treatment and the number of sessions (fewer *mean* sessions in FT: –1.44; $p = 0.064$) and strong evidence (step 3) that the number of therapy sessions was associated with the risk of self-harm during follow-up (HR 1.02; $p < 0.0001$), with risk increasing with more sessions. When the number of sessions attended is accounted for, there is weak evidence of a direct treatment effect (step 3) with an increased risk of self-harm in FT (HR 1.29; $p = 0.083$).

There was strong evidence (step 2) of an association between treatment group and the length of experience of the lead therapist (more experienced in FT: OR 2.38; $p=0.0003$), but no evidence that length of experience was associated with the risk of self-harm (step 3).

For both the number of sessions attended and the use of psychotropic medication, although there is no strong evidence of a treatment effect in steps 1 or 3, the magnitude of the treatment coefficient increased in step 3 and the relationship between treatment, mediator, and self-harm outcome warrants further investigation

Table 8: Mediators analysis: Assessment of the Baron and Kenny mediation steps[a]

		Step 1 $Y = h_0(t)\exp(\beta_{10} X)$		Step 2 $Me = \beta_{20} + \beta_{21} X$		Step 3 $Y = h_0(t)\exp(\beta_{31} X + \beta_{32} M)$			
		**Total effect of Rand on Outcome (β_{10})**		**Effect of Rand on Mediator (β_{21})**		**Effect of Mediator on Outcome (β_{32})**		**Direct effect of Rand on Outcome (β_{31})**	
**Process mediator variable**	**N**	**Hazard Ratio (95% CI)**	**p-value**	**Estimate (95% CI)**	**p-value**	**Hazard Ratio (95% CI)**	**p-value**	**Hazard Ratio (95% CI)**	**p-value**
Young person on any psychotropic medications	774	1.19 (0.90, 1.58)	0.22	OR: 0.60 (0.39, 0.91)	0.016	2.10 (1.47, 3.00)	<0.0001	1.26 (0.95, 1.67)	0.12
Years spent working in CAMHS by lead therapist	487	1.30 (0.88, 1.93)	0.19	OR: 2.38 (1.49, 3.82)	0.0003	0.78 (0.46, 1.33)	0.37	1.35 (0.90, 2.02)	0.15
Number of sessions attended	787	1.20 (0.91, 1.59)	0.20	-1.44 (-2.95, 0.08)	0.064	1.02 (1.01, 1.03)	<0.0001	1.29 (0.97, 1.71)	0.083

[a] The effect of Rand is in reference to FT compared to TAU (TAU is the reference category). For psychotropic medications the reference category is 'No' hence the effect of the mediator is in relation to Yes: Young Person was on psychotropic medications during follow up; and for years spent working in CAMHS the reference category is less than 4 years hence the effect of the mediator is in relation to YP with a lead therapist working in CAMHS for 4 or more years.

6. Comparison of baseline scores with population data

To contextualise the baseline scores, there is good data on a UK population that 10% of children aged 5-15 years score above the clinical threshold on the SDQ (compared with 66.2% in our sample)[8]. For depression, most estimates from large epidemiological studies are between 2% and 10% depending on age and severity[9]. These studies tend to use diagnostic interviews. However, the CDRS was designed to correlate reasonably well with diagnostic interviews and in our sample, gave a prevalence of 65.7%.
There are fewer population norms available for the FAD but in one study of adults with a range of different psychiatric diagnoses, only 26.1% of families in the control group scored above the clinical cut point, compared with 61.1–89.5% for families with a member with psychiatric disorder[10]. Our sample reported 84.7% above the cut point on the general functioning sub-scale of the FAD.

7. Health Economics: Intervention costs

Table 9: Average intervention costs by trial arm

Type of service		TAU (N=388)	FT (N=394)
CAMHS services	Mean (SD)	£800.7(£1,412.7)	N/A
	Min	£0.0	N/A
	Max	£18,103.5	N/A
Qualified family therapists	Mean (SD)	N/A	£2,075.5 (£1,506.3)
	Min	N/A	£0.0
	Max	N/A	£9,266.0
Telephone contact	Mean (SD)	£56.1 (£219.7)	£59.6 (£255.9)
	Min	£0.0	£0.0
	Max	£3,640.0	£4,095.0
Therapist's supervision	Mean (SD)	£18.5 (£46.9)	£48.1 (£60.5)
	Min	£0.0	£0.0
	Max	£373.4	£438.7

Table 10: Total costs of NHS resources used by trial arm with multiple imputations*

		TAU (N=388)	FT (N=394)	p-value (Mann-Whitney test)
**Total NHS costs^** (including *actual intervention costs)*	Mean	£3,725.5	£4,991.70	<0.0001
	(SD)	(£3,786.0)	(£3,766.9)	
	Median	£2,750.0	£3,976.6	
	Min	£164.0	£403.4	
	Max	£29,215.9	£32,085.2	
**Total *actual intervention costs*^^**	Mean	£875.3	£2,183.2	<0.0001
	(SD)	(£1,471.2)	(£1,558.8)	
	Median	£409.6	£1939.5	
	Min	£0.0	£0.0	
	Max	£18,683.5	£9,350.0	
**Total health and social services costs**	Mean	£1,403.8	£1,259.1	0.12
	(SD)	(£1,760.4)	(£1,462.8)	
	Median	£813.7	£722.8	
	Min	£0.0	£0.0	
	Max	£15,650.0	£12,451.2	
**Total hospital services costs from NHS Digital records** (inpatient stays and accident and emergency visits)^^	Mean	£1,335.9	£1,412.9	0.29
	(SD)	(£2,217.4)	(£2,550.8)	
	Median	£812.5	£810.2	
	Min	£0.0	£0.0	
	Max	£15,390.4	£24,190.2	
**Total reported hospital outpatient visits costs**	Mean	£117.7	£136.7	0.78
	(SD)	(£325.8)	(£444.6)	
	Median	£5.5	£4.5	
	Min	£0.0	£0.0	
	Max	£3,852.8	£7,194.4	
**Total costs of appointments** post-randomisation but before first treatment appointment^^	Mean	N/A	£5.3	N/A
	(SD)	N/A	£39.8	
	Median	N/A	£28.0	
	Min	N/A	£0.0	
	Max	N/A	£575.0	
**Medication costs^^**	Mean	£0.7	£3.3	0.71
	(SD)	(£11.0)	(£60.5)	
	Median	£0.0	£0.0	
	Min	£0	£0	
	Max	£214.8	£1,197.1	

*Include estimated costs after imputation – these figures were used in the cost-utility analyses.

^The cost of appointments that took place after randomisation but before the first treatment appointment in the FT arm is also included. ^^These costs were not imputed.

8. Health Economics: Monte Carlo simulation, model parameters

Health economic evaluation studies aim to assess health strategies in terms of their cost-effectiveness and inform public policies. While the within-trial analysis provides an evaluation of the cost-effectiveness restricted to the follow-up horizon of 18 months, the NHS is interested to understand the cost and the consequences between competing treatments beyond the trial follow-up. A longer time horizon will reflect all important differences in costs or consequences between FT and TAU. Decision analysis modelling is key in this context and consists in extrapolating the costs and the consequences using probabilities and assumptions based on the data collected in a trial and literature data extraction. Markov models are recursive (repetitive) decision trees that are used for modelling conditions that have events that may occur repeatedly over time.
The model (figure HE.1) included three possible health states: self-harm (SH) defined as self-harming at least once in a period of six months, no self-harming (noSH), and death. Markov models describe participant progression over time through a pathway of health states, with movement between the health states being triggered by events - in this case self-harm events or death.

Figure 2: Three-state Markov model (self-harm – SH, no self-harming - noSH)

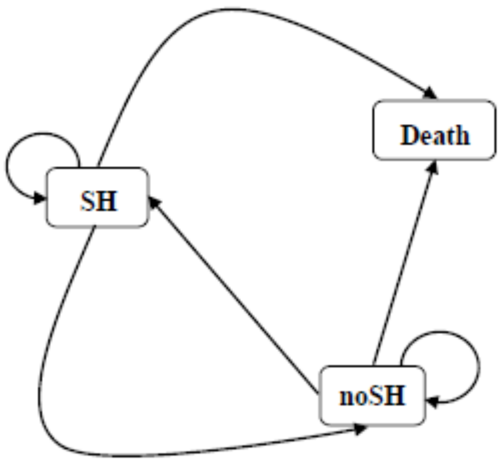

The model inputs were derived from the trial data. Specifically, the proportion of the participants beginning in each health state in the model was derived directly from the proportion of participants in the trial who remained in the SH state or moved to noSH. In the first cycle of the model, all participants started from SH in both arms and this was informed by the inclusion criteria for the trial. No participants died over the 18 months of trial follow-up but, to account for possible death, it was assumed that the probability of a participant moving from SH to death or from noSH to death was minimal and equal to 0.0001, regardless of the trial arm. Derivation of the post-18-month transition rates between the different states required extrapolation beyond the follow-up period of the trial.

Any intervention costs were assumed to occur equally over the first 12 months for each arm based on the trial data. Since, it was not possible to distinguish whether these costs were incurred only by those in the SH state, in the noSH state, or both, it was assumed to be the same for any of the states.
Resource use and costs were associated with each health state and participants' accumulated costs and health benefits in each state over 6-month cycles. Participant cost and utility data were available at 6 months, 12 and 18 months from the trial data and were directly included into the model to estimate longer term costs and health benefits. Death was assumed to be associated with a zero utility and zero cost.
The full list of the model parameters and distributions applied in the model is given in Table HE.3, with the chosen distributions being based on the observed variance data.

Table 11: Markov input parameters (self-harm – SH, no self-harming – noSH, YP - Young People)

		Mean	Distribution	Standard error	Source
**Global parameters**	Discount rate	0.035	Fixed		NICE guidance
**Health state costs in TAU arm (6 months)**	SH	£1,182	Lognormal	£1,493	
**Health state costs in TAU arm (12 months)**	SH	£1,698	Lognormal	£1,628	
	noSH	£709	Lognormal	£1,116	
	Death	0	Fixed	-	
**Health state costs in TAU arm (18 months)**	SH	£1,510	Lognormal	£1,022	
	noSH	£817	Lognormal	£1,432	
	Death	0	Fixed	-	
**Health state costs in FT arm (6 months)**	SH	£1,049	Lognormal	£1,482	
**Health state costs in FT arm (12 months)**	SH	£2,186	Lognormal	£2,198	
	noSH	£763	Lognormal	£1,228	
	Death	0	Fixed	-	
**Health state costs in FT arm (18 months)**	SH	£2,530	Lognormal	£2,282	
	noSH	£649	Lognormal	£1,054	
	Death	0	Fixed	-	
**Health state utilities in TAU arm (6 months)**	SH	0.760	Beta	0.161	
**Health state utilities in TAU arm (12 months)**	SH	0.751	Beta	0.187	
	noSH	0.784	Beta	0.180	
	Death	0	Fixed	-	
**Health state utilities in TAU arm (18 months)**	SH	0.754	Beta	0.033	
	noSH	0.808	Beta	0.157	SHIFT trial data
	Death	0	Fixed	-	
**Health state utilities in FT arm (6 months)**	SH	0.799	Beta	0.178	
**Health state utilities in FT arm (12 months)**	SH	0.793	Beta	0.184	
	noSH	0.813	Beta	0.194	
	Death	0	Fixed	-	
**Health state utilities in FT arm (18 months)**	SH	0.732	Beta	0.239	
	noSH	0.823	Beta	0.179	
	Death	0	Fixed	-	
**Transition probabilities (at 6 months)**	Proportion YPs stopping SH (noSH) from SH in the TAU arm	0.858	Beta	0.0003	
	Proportion of YPs stopping SH (noSH) from SH in the FT arm	0.845	Beta	0.0003	
**Transition probabilities (at 12 months)**	Proportion of YPs stopping SH (noSH) from SH in the TAU arm	0.716	Beta	0.004	
	Proportion of YPs SH from noSH in the TAU arm	0.066	Beta	0.0002	
	Proportion of YPs stopping SH (noSH) from SH in the FT arm	0.803	Beta	0.003	
	Proportion of YPs SH from noSH in the FT arm	0.078	Beta	0.0002	

**Transition probabilities (at 18 months)**	Proportion of YPs stopping SH (noSH) from SH in the TAU arm	0.775	Beta	0.004
	Proportion of YPs SH from noSH in the TAU arm	0.063	Beta	0.0002
	Proportion of YPs stopping SH (noSH) from SH in the FT arm	0.684	Beta	0.006
	Proportion of YPs SH from noSH in the FT arm	0.095	Beta	0.0002

9. References

1. Carr A. The effectiveness of family therapy and systemic interventions for child-focused problems. *Journal of Family Therapy* 2009; **31**(1): 3-45.

2. Pote H, Stratton P, Cottrell D, Shapiro D, Boston P. Systemic family therapy can be manualised: research process and findings. *Journal of Family Therapy* 2003; **25**: 236-62.

3. Boston P, Eisler I, Cottrell D. Systemic Family Therapy Manual for adolescent Self-Harm. Leeds: University of Leeds; 2009. https://medhealth.leeds.ac.uk/info/645/psychiatry_and_behavioural_sciences/1365/leeds_family_therapy_and_research_centre Downloaded 05/04/2007

4. Masterson C, Barker C, Jackson D, Boston P. Constructing a SHIFT adherence measure (SAM): the development of a family therapy integrity measure for the SHIFT trial. *Journal of Family Therapy* 2016; **38**: 274-90.

5. Schafer JL. Analysis of incomplete multivariate data. Boca Raton: CRC press; 1997.

6. Rubin DB. Multiple Imputation for Nonresponse in Surveys. . New York: Wiley; 1987.

7. Baron RM, Kenny DA. The moderator-mediator variable distinction in social psychological research: conceptual, strategic and statistical considerations. *Journal of Personality and Social Psychology* 1986; **51**: 1173-82.

8. Meltzer H, Gatward R, Goodman R, et a. Mental Health of Children and Adolescents in Great Britain. London: Stationery Office; 1999.

9. Fonagy P, Cottrell D, Phillips J, Glaser D, Allison E. Depressive Disorders. In What Works for Whom? A Critical Review of Treatments for Children and Adolescents. 2nd ed. New York: Guilford; 2014.

10. Ryan CE, Epstein NB, Keitner GI. Evaluating and treating families: The McMaster approach: Taylor & Francis; 2005.
